# Utilization of Cyanoacetohydrazide and Oxadiazolyl Acetonitrile in the Synthesis of Some New Cytotoxic Heterocyclic Compounds

**DOI:** 10.3390/molecules21020155

**Published:** 2016-01-29

**Authors:** Soheir A. Shaker, Magda I. Marzouk

**Affiliations:** Chemistry Department, Faculty of Science, Ain Shams University, Abassia 11566, Cairo, Egypt; soheirshaker@yahoo.com

**Keywords:** cyanoacetohydrazide, oxadiazolylacetonitrile, pyridazinone

## Abstract

A (pyridazinyl)acetate derivative was reacted with thiosemicarbazide and hydrazine hydrate to yield spiropyridazinone and acetohydrazide derivatives, respectively. The acetohydrazide derivative was used as a starting material for synthesizing some new heterocyclic compounds such as oxoindolinylidene, dimethylpyrazolyl, methylpyrazolyl, oxopyrazolyl, cyanoacetylacetohydrazide and oxadiazolylacetonitrile derivatives. The behavior of the cyanoacetylacetohydrazide and oxadiazolylacetonitrile derivatives towards nitrogen and carbon nucleophiles was investigated. The assigned structures of the prepared compounds were elucidated by spectral methods (IR, ^1^H-NMR ^13^C-NMR and mass spectroscopy). Some of the newly prepared compounds were tested *in vitro* against a panel of four human tumor cell lines, namely hepatocellular carcinoma (liver) HePG-2, colon cancer HCT-116, human prostate cancer PC3, and mammary gland breast MCF-7. Also they were tested as antioxidants. Almost all of the tested compounds showed satisfactory activity.

## 1. Introduction

**Figure 1:**
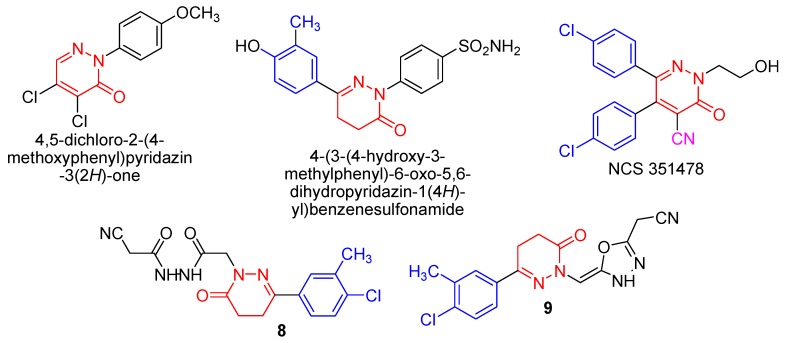
Anticancer pyridazine derivatives.

Living organisms have difficulties in the construction of N-N bonds that limits the natural abundance of compounds having such bonds. Pyridazinone derivatives, a class of compounds containing the N-N bond, exhibit a wide range of pharmacological activity [1], including analgesic, antidepressant, anti-inflammatory [2,3,4,5,6], antimicrobial [7,8] as well as herbicidal activities [9]. There are numerous reports available in the literature, which indicate the potential anticancer effects of pyridazinones. 6-(4-Hydroxy-2-methylphenyl)-2-(*p*-sulfamylphenyl)-4,5-dihydropyridazine-3(2*H*)-one [10] showed high activity against HL-60 (TB) (leukemia), SR (leukemia), NCIH522 (non-small-cell lung cancer), and BT-549 (breast cancer), the *p*-methoxydichloropyridazone [11] displayed a good inhibition of tumour growth in mice for the resistant MAC16 cell line. Some diphenylpyridazine derivatives [12] (particularly NSC 351478) were effective in the treatment of P388 leukemia in mice. The substituents at position 2 of the pyridazinone ring do not fall into a clear pattern; alterations at this position can have major effects on the activity of the resulting compounds. More specific was the effect of chlorinating the phenyl rings. Position 4′ seems most important, but a second chlorine at position 3′ further enhances inhibition of microtubule assembly *in vitro* (Figure 1). Further investigations will be required for a more detailed evaluation of anticancer pyridazine compounds with different molecular mechanisms for enhancing anticancer activities and minimizing toxicities. For these reasons we have introduced hydrazide and 1,3,4-oxadiazole moieties to the pyridazine ring to enhance the cytotoxic activity.

Hydrazides are very useful starting materials for the construction of several heterocyclic compounds such as 1,3,4-oxadiazoles [13], 1,3-thiazoles [14], 1,3,4-thiadiazoles [15], 1,2,4-triazoles [16], 1,2,4-triazolo[3,4-*b*]-1,3,4-thiadiazoles [17] 1,2,4-triazolo[3,4-*b*]-1,3,4-thiadiazines [18], pyrroles [19] and pyrazoles [20]. The common practical route for hydrazide synthesis is the treatment of esters with hydrazine hydrate. They are also synthesized by the reaction of hydrazine hydrate or its derivatives with carboxylic acids and acyl halides [21]. Hydrazides including α,β-unsaturated acids were synthesized from the reaction of activated esters and/or amides with hydrazines [22]. Hydrazide oligonucleotides were also synthesized [21]. The use of microwave irradiation is a facile way of preparing hydrazides by solvent-free reactions of acid derivatives with hydrazine hydrate [23,24,25]. Among several commercially available substituted hydrazines, cyanoacetic acid hydrazide has received the most attention [26,27]. Cyanoacetic acid hydrazide was obtained by the addition of hydrazine hydrate to ethyl cyanoacetate in methanolic ice-cooled solution [28]. Cyanoacetic acid hydrazide is a versatile and convenient intermediate for the synthesis of wide variety of heterocyclic compounds. This substrate can act as an ambident nucleophile, that is, as both a *N*- and *C*-nucleophile. The reactions of cyanoacetic acid hydrazide with numerous reactants (nucleophiles and electrophiles) are used in the synthesis of a variety of polyfunctional heterocyclic compounds with pharmacological interest.

**Figure 2:**
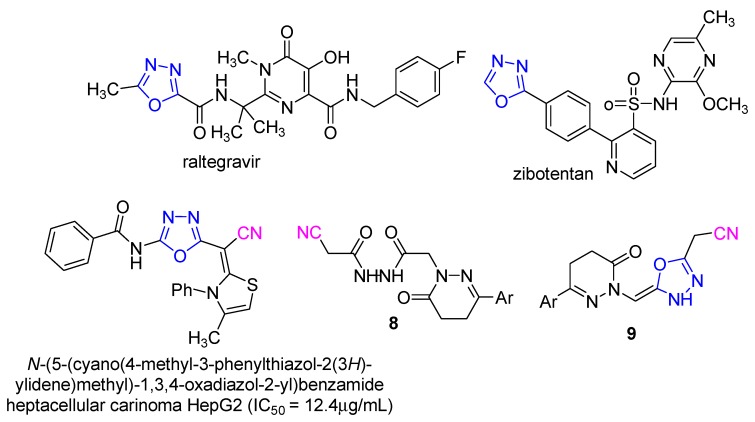
1,3,4-Oxadiazole drugs and compounds **8** and **9**.

1,3,4-Oxadiazoles constitute an important family of heterocyclic compounds as they have attracted significant interest in medicinal chemistry, pesticide chemistry and polymer science. The 1,3,4-oxadiazoles have been found to exhibit diverse biological activities such as antimicrobial [29,30,31,32,33], antitubercular [34], antioxidant [35], antimalarial [36], analgesic [37], anti-inflammatory [38,39], anticonvulsant [40], hypoglycemic [41] activities, as well as other biological properties such as genotoxic [42] and lipid peroxidation inhibitory activities [43]. Two examples of compounds containing the 1,3,4-oxadiazole ring used in clinical medicine are raltegravir, an antiretroviral drug [44] and zibotentan, an anticancer agent [45] (Figure 2). Also *N*-(5-(cyano(4-methyl-3-phenylthiazol-2(3*H*)-ylidene)methyl)-1,3,4-oxadiazol-2-yl)benzamide which contains the 1,3,4-oxadiazole ring and the nitrile group showed antitumor activity [46].

1,3,4-Oxadiazole derivatives were synthesized via the reaction of hydrazide derivatives using different reagents such as CS_2_ [47], POCl_3_/aromatic carboxylic acid [48], thionyl chloride [49,50,51], triphenylphosphine oxide, [52] silica-supported dichlorophosphate [53] and ZrCl_4_ [54]. The syntheses of 1,3,4-oxadiazoles via the Huisgen reaction [55,56] is a less popular methodology than the methods mentioned above. On the other hand, Efimova *et al.* reported [57] the synthesis of 1,3,4-oxadiazole derivatives via the acylation of tetrazoles with acetic and benzoic anhydrides. The yield of the products obtained by these methods ranged from 73%–96%.

In the light of these facts, and as a continuation of our efforts towards synthesizing biologically active heterocyclic compounds especially those with antitumor activity [58,59,60,61,62], we planned to synthesize new hydrazide and 1,3,4-oxadiazole derivatives in the hope that new antitumor agents might be discovered.

## 2. Results and Discussion

### 2.1. Chemistry

**Scheme 1:**
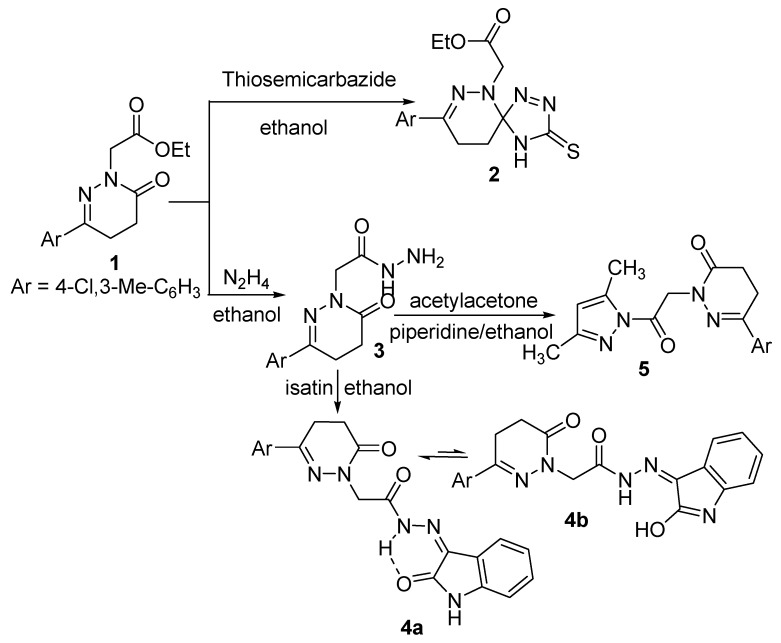
Synthetic route for the preparation of compounds **2**–**5**.

The ester derivative **1** was reacted with nitrogen nucleophiles such as thiosemicabazide and hydrazine hydrate to yield the spiro compound **2** [63] and the acetohydrazide derivative **3**, respectively (Scheme 1). The IR spectrum of compound **2** showed bands corresponding to NH and C=O groups at 3263 and 1741 cm^−1^, respectively. The ^1^H-NMR spectrum is in accord with the proposed structure, as it showed signals for NH, aliphatic and aromatic protons, four CH_2_ and two CH_3_ moieties at δ 8.58, 7.40–7.77, 4.36, 4.14, 2.98, 2.56, 2.37 and 1.20 ppm, respectively. The presence of the triplet and the quartet signals at δ 1.20 and 4.14 ppm, respectively, in its ^1^H-NMR spectrum and also the presence of signals at δ 14.03 and 60.76 ppm in its ^13^C-NMR spectrum indicated the presence of the ester group which proved that the reaction occurred at the carbonyl carbon of the ring rather than that of the ester. The ^13^C-NMR spectrum showed a signal at δ 94.28 ppm which supported the spiro structure. Further evidence was gained from the mass spectrum as it showed the correct molecular ion peak at *m*/*z* 379, in addition to some other important fragment peaks.

The formation [63] of the spiro derivative **2** could be explained on the basis of nucleophilic attack of the nitrogen atom of the thiosemicarbazide molecule on the ring carbonyl carbon atom followed by ring closure through the elimination of water and dehydrogenation.

The acetohydrazide **3** was reacted with different carbon nucleophiles to yield some new interesting heterocyclic compounds. It was reacted with cyclic and acyclic diones such as isatin and acetylacetone to yield the corresponding oxoindolinylidene **4** and the pyrazolyl **5** derivatives, respectively. The structures of compounds **4** and **5** were confirmed by their analytical and spectroscopic data. The IR spectrum of compound **4** showed broad band at 3173 cm^−1^ assigned to N-H and bands at 1642–1695 cm^−1^ assigned to 3C=O, and a band at 1619 cm^−1^ for C=N. The lower absorption value for the indolyl carbonyl suggests the existence of compound **4** as its chelated form **4a** as shown (Scheme 1). Inspection of the ^1^H-NMR spectrum of compound **4** showed the existence of three exchangeable broad singlet signals in the downfield region at δ 11.21, 12.57 and 13.21 corresponding to 2NH and OH protons, this suggests the existence of compound **4** in deuterated dimethyl sulfoxide solution as an equilibrium mixtures of **4a,b** in the ratio of 72:27 as shown (Scheme 1). Compound **4** is stabilized by conjugation and intramolecular hydrogen bonding. Further evidence was gained from mass spectrum as it showed the correct molecular ion peak at *m*/*z* 423 beside some other important peaks. The ^13^C-NMR spectrum was also in accordance with the proposed structure.

The IR spectrum of compound **5** showed two bands attributed to 2C=O at 1731 and 1681 cm^−1^, respectively. The ^1^H-NMR spectrum of compound **5** showed signals at 7.40–7.78, 6.25, 5.22, 3.05, 2.61 ppm corresponding to aromatic protons, =CH and three CH_2_, respectively. The presence of signals at 2.49, 2.45 and 2.21 ppm also indicated the presence of three CH_3_ groups. In addition, the ^1^H-NMR spectrum was devoid of any signals corresponding to NH and NH_2_ protons which are in accord with the proposed structure. The ^13^C-NMR spectrum exhibited signals at δ 13.45, 13.63 and 19.54 ppm due to the presence of three CH_3_ groups. The mass spectrum of compound **5** showed the molecular ion peak at *m*/*z* 358, which is coincident with its molecular weight.

**Scheme 2:**
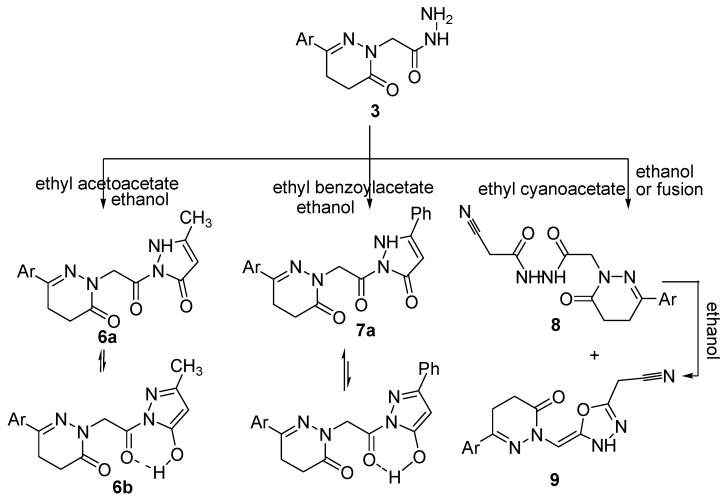
Synthetic route for the preparation of compounds **6**–**9**.

The reaction of the acetohydrazide **3** with ethyl acetoacetate and ethyl benzoylacetate yielded the corresponding pyrazolone derivatives **6** and **7**, respectively (Scheme 2). The reactions occurred at the ketonic carbonyl followed by 5-*exo-trig* ring closure. The structures of compounds **6** and **7** were confirmed by analytical and spectroscopic data. The IR spectrum of compound **6** showed bands for NH and C=O groups at 3195, 1675, 1661 and 1642 cm^−1^, respectively. Further evidence for the structure of compound **6** was gained from its ^1^H-NMR spectrum which showed signals at 9.98, 7.43–7.75, 4.41, 3.00, 2.58, 2.72 and 2.35 ppm attributed to OH, aromatic protons, three CH_2_ and two CH_3_ groups, respectively. The higher δ signal at 9.98 ppm for the exchangeable broad singlet and the presence of a signal at δ 135.71 ppm in its NMR (^1^H and ^13^C) spectra are good evidence for the existence of compound **6** in deuterated DMSO solution as its hydroxypyrazole **6b**. Meanwhile, the IR spectrum of compound **7** showed bands for NH and C=O groups at 3312, 1669 and 1648 cm^−1^, respectively. The ^1^H-NMR spectrum of compound **7** showed signals at 9.09, 7.40–7.75, 4.31, 4.24, 3.01, 2.55, and 2.36 ppm attributed to OH, aromatic protons, CH_2_, NH, two CH_2_ and a CH_3_, respectively. However, the appearance of two exchangeable broad singlet signals, one in the upfield region and the second in the downfield region in the ratio of 35:65 suggests the existence of compound **7** as an equilibrium mixture of **7a** and **7b** (Scheme 2). The ^13^C-NMR spectrum was also in accordance with the proposed structure. Further evidence for compounds **6** and **7** was gained from mass spectra, as they showed the correct molecular ion peaks for compounds **6** and **7** at *m*/*z* 308 and 422, respectively, beside some other important peaks.

The cyanoacetylacetohydrazide **8** was formed as a sole product upon refluxing equimolar amounts of an alcoholic solution of the acetohydrazide **3** with ethyl cyanoacetate, while fusion of the acetohydrazide **3** with excess of ethyl cyanoacetate yielded both the open chain product cyanoacetyl acetohydrazide derivative **8** and the cyclic oxadiazolyl acetonitrile derivative product **9**. The structures of these compounds were confirmed by analytical and spectroscopic data. The IR spectra of compounds **8** and **9** revealed the existence of the cyano groups at 2212 and 2206 cm^−1^, respectively. The ^1^H-NMR spectrum of compound **8** showed a singlet signal corresponding to the CH_2_CN group at δ 4.33 ppm, and two singlets at δ 9.44 and 9.89 ppm corresponding to two NH protons. The ^1^H-NMR spectrum of compound **9** meanwhile showed a singlet signal for the CH_2_CN group at δ 4.41 ppm, a multiplet for HC=C and aromatic protons at δ 7.38–7.75 ppm and a singlet for one NH at δ 9.99 ppm. The ^13^C-NMR spectrum of compound **9** showed signals at δ 19.60, 60.75, 120.14 and 172.18 ppm corresponding to CH_2_, HC=C, C≡N and HC=C, respectively. Further evidence was gained from the mass spectra of compounds **8** and **9**, as they showed the correct molecular ion peaks at *m*/*z* 361 and 343, respectively, in addition some other important fragmentation peaks. The presence of C≡N in the IR as well as ^13^C-NMR spectra and also the presence of the CH_2_CN in the NMR (^1^H and ^13^C) spectra supported the proposed structures of compounds **8** and **9**. Chemical proof for the structure of compound **9** was gained by heating compound **8** in ethanol to afford the oxadiazolyl acetonitrile derivative **9**. Compound **8** was formed via Claisen condensation of the terminal amino group with ester group of the ethyl cyanoacetate, while compound **9** is formed from compound **8** through keto-enol tautomerism followed by cyclization. Further evidence for the structures of compounds **8** and **9** were obtained through studying their chemical reactivity towards some chemical reagents.

**Scheme 3:**
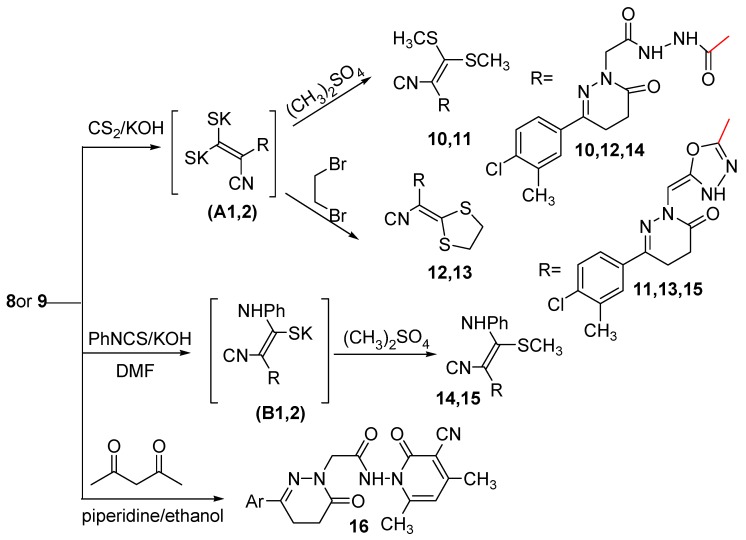
Synthetic route for the preparation of compounds **10**–**16**.

Compounds with an activated methylene group react as carbanions in the presence of a base with the electrophilic carbon disulfide to give dithiocarboxylates which can be converted to ketene dithioacetals on treatment with an excess of the alkylating reagent. Thus, stirring of the cyanoacetyl acetohydrazide **8** and the oxadiazolyl acetonitrile **9** with carbon disulfide in the presence of KOH in DMF followed by the addition *in situ* of dimethyl sulfate afforded compounds **10** and **11** via the intermediates (A1,2) (Scheme 3).

Also cyclobromination of the intermediates (A1,2)with dibromoethane afforded compounds **12** and **13**, respectively. The reaction proceeded via nucleophilic addition of the carbanions on CS_2_ to form the potassium salt intermediates (A1,2) followed by *in situ* cyclization through a S_N_2 mechanism to yield the cyclic compounds **12** and **13**, respectively. The structures of compounds **10**–**13** were established on the basis of analytical and spectral data. The IR spectra of compounds **10**–**13** showed bands characteristic for NH, CN and C=O groups in the range 3436–3200, 2214–2204 and 1677–1642 cm^−1^, respectively. Their ^1^H-NMR spectra are in accord with the suggested structures, where they are devoid of a signal corresponding to CH_2_CN protons. However, they displayed signals related to two CH_3_S protons for compounds **10** and **11** at δ 2.89, 2.88 ppm, respectively, and SCH_2_CH_2_S protons for compounds **12** and **13** at δ 3.15 and 4.41 ppm, respectively. The ^13^C-NMR spectra of compounds **12** and **13** exhibited signals at δ 34.28 and 53.80 ppm, respectively indicating the presence of S-CH_2_-CH_2_-S. Further evidence was gained from mass spectra as they showed the correct molecular ion peaks for compounds **11**–**13** at *m*/*z* 447, 463 and 445, respectively beside some other important peaks.

Furthermore, reaction of compounds **8** and **9** with phenyl isothiocyanate in the presence of KOH yielded the potassium salt intermediates) (B1,2). Alkylation of the potassium salt intermediates (B1,2) *in situ* with dimethyl sulfate gave compounds **14** and **15**, respectively. The structures of compounds **14** and **15** were elucidated on the basis of the elemental analyses and spectral data. The IR spectra of compounds **14** and **15** displayed bands corresponding to NH, CN and C=O groups at 3182, 3333, 3211, 2203, 2202, 1673 and 1714 cm^−1^, respectively. Further support for the assigned structures of compounds **14** and **15** was gained from their ^1^H-NMR spectra. The ^1^H-NMR spectrum of compounds **14** showed characteristic signals for three NH, aromatic protons, three CH_2_ and two CH_3_ at 10.59, 10.27, 10.07, 6.92–8.71, 4.41, 3.00, 2.75, 2.58 and 2.36 ppm, respectively, while, the ^1^H-NMR spectrum of compounds **15** showed characteristic signals for two NH, aromatic protons, =CH, two CH_2_ and two CH_3_ at 8.62, 7.57, 7.27–7.73, 6.96, 4.41, 3.00, 2.67, 2.41 and 2.34 ppm, respectively. The presence of an extra NH and CH_3_ protons and also the absence of the CH_2_CN protons supported the proposed structures of compounds **14** and **15**. The ^13^C-NMR spectra were also in accordance with the proposed structures as they showed the presence of two CH_3_ and twelve aromatic carbons.

The cyanoacetyl acetohydrazide **8** was reacted with acetylacetone in piperidine to give **16**. Compound **8** has two sites that have acidic hydrogens which can react with the carbonyl carbon of acetylacetone. The acetonitrile carbanion is more stabilized by the strong electron attracting character of both the C=O and C≡N groups. The structure of compound **16** was elucidated on the basis of the elemental analysis and spectral data. The IR spectrum showed three bands at 1677, 1657 and 1642 cm^−1^ assignable to three C=O groups and also bands at 3180 and 2214 cm^−1^ assignable to NH and C≡N groups, respectively, while the ^1^H-NMR spectrum of compound **16** showed signals at δ 2.28 and 2.35 and 9.99 ppm attributed to three CH_3_ and one NH, respectively. The presence of one NH and three CH_3_ protons and also the absence of the CH_2_CN protons supported the proposed structure of compound **16**. The ^13^C-NMR spectrum indicated the presence of three methyl groups.

**Scheme 4:**
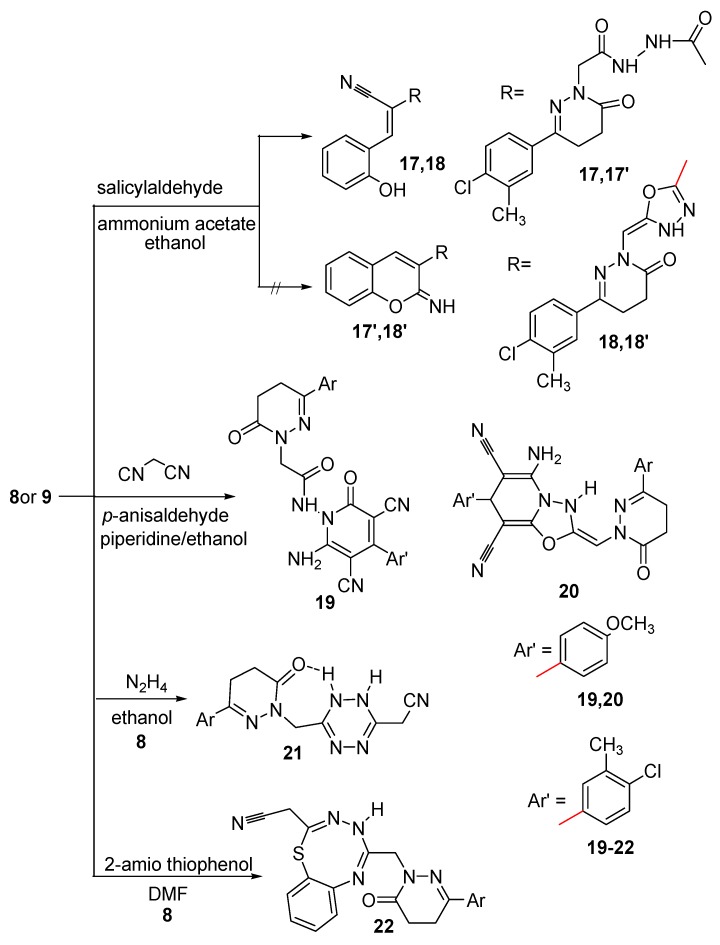
Synthetic route for the preparation of compounds **17**–**22**.

Condensation of compounds **8** and **9** with salicylaldehyde in boiling ethanol and in the presence of ammonium acetate afforded the corresponding hydroxyphenyl derivatives **17** and **18**, respectively (Scheme 4). The structures of compounds **17** and **18** were elucidated on the basis of their elemental analyses and spectral data. The IR spectra of compounds **17** and **18** showed bands for OH, NH in the range 3188–3419 cm^−1^ and also showed signals attributed to C≡N groups in the range 2206–2207 cm^−1^, which indicated the formation of the open structures **17** and **18** and not the cyclic structures **17′** and **18′**. The ^1^H-NMR spectrum of compound **17** showed signals corresponding to OH, aromatic, =CH and two NH protons at 7.18, 7.38–7.75, 8.41 and 9.99 ppm, respectively, beside other signals corresponding to three CH_2_ and CH_3_ at 4.41, 3.01, 2.58 and 2.34 ppm, respectively. The ^1^H-NMR spectrum of compound **18** showed signals corresponding to OH, aromatic, =CH and NH protons at 7.45–7.75 and 9.97 ppm, respectively, beside other signals corresponding to 2CH_2_ and CH_3_ at 2.99, 2.72 and 2.35 ppm, respectively. The ^13^C-NMR spectrum was also in accordance with the proposed structures of compounds **17** and **18**. The presence of the C≡N in the IR spectra and the OH proton in the ^1^H-NMR spectra indicated the formation of the open structures **17** and **18** not the cyclic structures **17′** and **18′**. Further evidence was gained from mass spectra as they showed the correct molecular ion peaks for compounds **17** and **18** at *m*/*z* 465 and 447, respectively, beside some other important peaks.

The pyridazinylacetamide derivatives **19** and **20** were synthesized via multicomponent reaction of compounds **8** and **9** with *p*-anisaldehyde and the active methylene compound malononitrile. The structures of compounds **19** and **20** were deduced from studying their spectroscopic data. The IR spectrum of compound **19** revealed bands at 3457, 3271, 3178 cm^−1^ assignable to NH and NH_2_, in addition to three bands in the 1676–1623 range and a band at 2206 cm^−1^ assignable to C=O and C≡N, respectively. The ^1^H-NMR spectrum of compound **19** showed signals attributable to NH_2_ and NH at δ 9.87 and 10.00 ppm, respectively, and a signal at δ 3.87 ppm assignable to OCH_3_ protons, plus signals for aliphatic and aromatic protons. The IR spectrum of compound **20** revealed bands for NH, NH_2_, C≡N and C=O at 3448, 3178, 2207 and 1674 cm^−1^, respectively. The ^1^H-NMR spectrum of compound **20** showed signals attributable to NH and NH_2_ at δ 8.40 and 2.72 ppm, respectively, and also signals at δ 6.95–7.71, 5.07, 4.41, 3.74, 3.02, 2.60 and 2.27 ppm assignable to aromatic, =CH, CH, OCH_3_, two CH_2_ and CH_3_ protons, respectively. The presence of signals attributed to two C≡N and OCH_3_ in the ^13^C-NMR spectra of compounds **19** and **20** supported the proposed structures. The presence of the NH_2_ in both the IR and ^1^H-NMR spectra and also the presence of OCH_3_ protons in the ^1^H-NMR spectra indicated the formation of compounds **19** and **20**. Further evidence was gained from mass spectra, which showed the correct molecular ion peaks for compounds **19** and **20** at *m*/*z* 543 and 527, respectively, in addition to some other important peaks.

Because tetrazines are of considerable interest, not only because of their inherent biological potential [64], but also because of their value as building blocks in synthetic transformations, compound **8** was condensed with hydrazine hydrate to afford the tetrazine derivative **21**. The elemental analysis and the spectroscopic data confirmed its structure. The IR spectrum revealed the existence of one band for one C=O at 1667 cm^−1^, beside bands corresponding to NH, and C≡N at 3312 and 2199 cm^−1^, respectively. The ^1^H-NMR spectrum of compound **21** showed signals corresponding to two protons of NH at 9.07 and 10.47 ppm, the higher value is due to hydrogen bonding, beside signals attributable to aromatic, six CH_2_ and CH_3_ protons at 7.35–7.71, 4.31, 4.26, 3.01, 2.55 and 2.36 ppm, respectively The ^13^C-NMR spectrum indicating the presence of 2C=N carbons of the tetrazine ring as it showed a signal at δ 163.40 ppm. Further evidence was gained from mass spectrum as it showed the correct molecular ion peak at *m*/*z* 357 and some other major peaks.

The thiatriazocinyl derivative **22** was synthesized via the reaction of compound **8** with 2-amino thiophenol. The IR spectrum showed bands at 3328, 3182 and 1660 cm^−1^ attributable to NH and C=O, and also it showed a band at 2205 cm^−1^ attributable to C≡N, which indicated that the nucleophilic attack did not occur at the C≡N group. The ^1^H-NMR spectrum showed signals attributable to NH, CH_2_N and CH_2_CN protons at 5.44, 4.39 and 2.95 ppm, beside the other signals for aliphatic and aromatic protons and was devoid of a signal attributable to NH_2_. The C=O group neighboring the CH_2_CN is more positive than the other C=O group, consequently, the reaction occurred at this carbonyl group rather than the other one, followed by ring closure to afford the desired compound **22**. Further evidence was gained from mass spectrum as it showed the correct molecular ion peak at *m*/*z* 450 beside some other important peaks.

### 2.2. Pharmacological Activity

#### 2.2.1. Antitumor Activity Using *in Vitro* Ehrlich Ascites Assay

**Figure 3:**
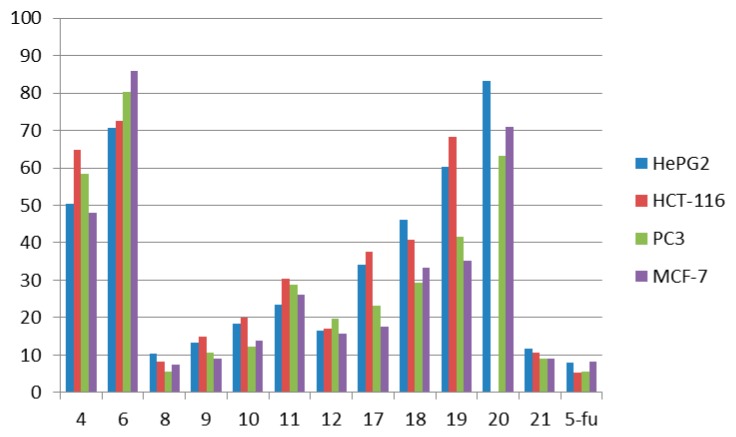
Cytotoxic activity of the tested compounds on different cell lines.

We assessed the cytotoxic action of the compounds (listed in Table 1 and shown in (Figure 3) against four human tumor cell lines namely: hepatocellular carcinoma (liver) HePG-2, colon cancer HCT-116, human prostate cancer cell line PC3 and mammary gland breast MCF-7.

**Table 1:** Cytotoxicity activity (IC_50_) of the tested compounds on different cell lines.

Comp. No.		*In Vitro* Cytotoxicity	IC_50_ (μg/mL) ^a^	
	HePG2	HCT-116	PC3	MCF-7
**4**	50.3 ± 4.22	64.7 ± 4.11	48.1 ± 3.64	58.4 ± 3.67
**6**	70.7 ± 4.65	72.6 ± 4.51	86.0 ± 4.63	80.4 ± 4.75
**8**	10.3 ± 0.81	8.1 ± 0.35	7.4 ± 0.34	5.6 ± 0.30
**9**	13.2 ± 1.31	14.8 ± 1.53	9.1 ± 0.86	10.5 ± 1.04
**10**	18.4 ± 1.06	20.0 ± 1.96	13.7 ± 1.37	12.3 ± 1.08
**11**	23.4 ± 1.46	30.3 ± 2.64	26.2 ± 1.60	28.7 ± 1.83
**12**	16.5 ± 1.35	16.9 ± 1.14	15.7 ± 1.56	19.7 ± 1.76
**17**	34.1 ± 2.30	37.5 ± 2.67	17.5 ± 1.42	23.1 ± 1.51
**18**	46.0 ± 3.61	40.7 ± 2.63	33.3 ± 2.07	29.4 ± 2.00
**19**	60.3 ± 3.97	68.3 ± 3.88	35.3 ± 2.94	41.5 ± 2.43
**20**	83.2 ± 4.83	>100	70.9 ± 4.75	63.1 ± 3.89
**21**	11.8 ± 1.12	10.5 ± 0.89	8.9 ± 0.45	9.1 ± 0.87
**5-fu**	7.9 ± 0.28	5.2 ± 0.14	8.3 ± 0.25	5.5 ± 0.21

^a^ IC_50_ (μg/mL): 1–10 (very strong), 11–20 (strong), 21–50 (moderate), 51–100 (weak), above 100 (non-cytotoxic).

In general, activity was observed by all of these molecules ranged from very strong to non-cytotoxic. The best results were observed for compound **8** (very strong activity) with IC_50_ 10.3 ± 0.81, 8.1 ± 0.35, 7.4 ± 0.34 and 5.6 ± 0.30 μg/mL for HePG-2, HCT-116, PC-3 and for MCF-7 cell lines, respectively. Its activity towards MCF-7 cells is equal to that of 5-flurouracil (5-FU, 5.5 ± 0.21 μg/mL). Compound **21** showed very strong activity towards the HCT-116 cell line with IC_50_ (10.5 ± 0.89 μg/mL), the PC-3 cell line (8.9 ± 0.45), which is nearly equal to 5-flurouracil (5-FU, 5.5 ± 0.21 μg/mL) and MCF-7 cell line (9.1 ± 0.87), and it also showed strong activity towards the HePG-2 cell line (11.8 ± 1.12). Compound **9** showed very strong activity towards the PC-3 (9.1 ± 0.86) and MCF-7 cell lines (10.5 ± 1.04), and it also showed strong activity towards the HePG-2 and HCT-116 cell lines 13.2 ± 1.31 and 14.8 ± 1.53 μg/mL, respectively. Meanwhile, compounds **10** and **12** showed strong activity towards the four cell lines. Compound **10** showed IC_50_ 18.4 ± 1.06, 20.0 ± 1.96, 13.7 ± 1.37 and 12.3 ± 1.08 μg/mL for the HePG-2, HCT-116, PC-3 and MCF-7 cell lines, respectively. Also, compound **12** showed IC_50_ 16.5 ± 1.35, 16.9 ± 1.14, 15.7 ± 1.56 and 19.7 ± 1.76 μg/mL for the HePG-2, HCT-116, PC-3 and MCF-7 cell lines, respectively. The observed activities of compounds **6**, **4**, **11**, **18**, **19** and **20** ranged from moderate to non-cytotoxic, with IC_50_ values from 23.4 ± 10 to higher than 100. Finally, compound **17** showed strong activity towards the PC-3 cell line with IC_50_ 17.5 ± 1.42 and moderate activity towards the HePG-2, HCT-116 and MCF-7 cell lines with IC_50_ values of 34.1 ± 2.30, 37.5 ± 2.67 and 23.1 ± 1.51 μg/mL, respectively. The relative viability of cells (%) for the tested compounds is listed in Table 2 and Table 3. The relationship between surviving fractions and the tested compounds concentration was plotted to obtain the survival curves of the four cell lines (the relative viability of cells (%) curves for the tested compounds is shown in Supplementary Materials Figures S1–S13).

**Table 2:** Relative viability of cells (%) for **5-FU** and compounds **4**, **6**, **8**, and **9**–**11**.

Compounds	Conc. (µg/mL)	HePG-2	HCT-116	PC3	MCF-7
**5-FU**	100 µg/mL	8.6	7.2	8.1	7.7
	50 µg/mL	17.1	12.0	15.8	14.3
	25 µg/mL	24.0	19.3	22.5	21.5
	12.5 µg/mL	33.1	30.6	36.7	34.6
	6.25 µg/mL	56.8	48.9	55.2	47.4
	3.125 µg/mL	70.6	60.5	74.1	58.3
	1.56 µg/mL	88.7	73.4	92.5	76.9
**4**	100 µg/mL	37.9	42.7	36.1	41.1
	50 µg/mL	48.8	55.5	48.4	52.9
	25 µg/mL	63.0	67.2	62.5	64.6
	12.5 µg/mL	75.6	81.3	74.2	78.2
	6.25 µg/mL	93.1	94.9	95.6	99.3
	3.125 µg/mL	100	100	100	100
	1.56 µg/mL	100	100	100	100
**6**	100 µg/mL	44.5	45.7	49.3	46.8
	50 µg/mL	57.2	56.6	60.6	61.2
	25 µg/mL	70.4	71.3	78.1	73.3
	12.5 µg/mL	82.9	83.4	91.7	86.9
	6.25 µg/mL	98.8	96.5	100	100
	3.125 µg/mL	100	100	100	100
	1.56 µg/mL	100	100	100	100
**8**	100 µg/mL	13.0	8.3	8.4	7.0
	50 µg/mL	18.2	17.9	14.3	12.3
	25 µg/mL	25.8	25.1	22.5	19.5
	12.5 µg/mL	36.9	33.7	31.9	31.9
	6.25 µg/mL	68.5	56.8	52.6	50.6
	3.125 µg/mL	75.6	71.4	70.8	62.2
	1.56 µg/mL	97.4	89.3	91.1	74.4
**9**	100 µg/mL	18.4	16.2	8.1	13.1
	50 µg/mL	26.3	25.3	17.6	18.9
	25 µg/mL	35.1	36.4	26.8	27.4
	12.5 µg/mL	43.7	48.1	37.2	38.2
	6.25 µg/mL	61.0	67.5	55.5	66.7
	3.125 µg/mL	84.2	88.7	78.1	76.1
	1.56 µg/mL	100	100	96.0	98.5
**10**	100 µg/mL	23.1	20.1	19.0	15.1
	50 µg/mL	30.0	32.4	26.4	24.2
	25 µg/mL	41.5	42.8	35.9	35.1
	12.5 µg/mL	53.8	55.5	43.2	42.8
	6.25 µg/mL	67.3	71.2	65.3	59.9
	3.125 µg/mL	89.7	95.3	83.6	81.7
	1.56 µg/mL	100	100	100	100
**11**	100 µg/mL	24.8	29.1	26.6	25.9
	50 µg/mL	35.7	39.7	37.3	38.8
	25 µg/mL	47.4	52.5	49.2	50.1
	12.5 µg/mL	58.1	63.8	60.5	65.9
	6.25 µg/mL	72.3	77.9	74.1	78.6
	3.125 µg/mL	91.8	96.2	97.2	96.7
	1.56 µg/mL	100	100	100	100

**Table 3:** Relative viability of cells (%) for compounds **12**, and **17**–**21**.

Compounds	Conc. (µg/mL)	HePG-2	HCT-116	PC3	MCF-7
**12**	100 µg/mL	19.9	18.3	21.0	22.9
	50 µg/mL	27.2	26.8	29.2	31.2
	25 µg/mL	36.1	39.5	38.3	43.8
	12.5 µg/mL	51.3	50.4	47.5	54.2
	6.25 µg/mL	71.4	72.6	65.4	71.3
	3.125 µg/mL	89.5	90.7	86.7	88.5
	1.56 µg/mL	100	100	100	100
**17**	100 µg/mL	29.1	31.5	19.7	24.2
	50 µg/mL	42.2	45.2	27.5	35.5
	25 µg/mL	51.9	55.8	39.1	46.1
	12.5 µg/mL	71.3	69.7	51.6	57.3
	6.25 µg/mL	84.5	81.3	73.4	72.6
	3.125 µg/mL	100	99.4	92.4	96.8
	1.56 µg/mL	100	100	100	100
**18**	100 µg/mL	35.9	33.6	31.1	28.6
	50 µg/mL	48.1	45.5	42.5	39.5
	25 µg/mL	60.5	57.1	53.2	51.3
	12.5 µg/mL	72.8	70.0	65.4	62.9
	6.25 µg/mL	89.2	91.2	78.0	77.8
	3.125 µg/mL	100	100	99.3	96.4
	1.56 µg/mL	100	100	100	100
**19**	100 µg/mL	38.7	43.7	29.5	31.9
	50 µg/mL	53.2	56.5	42.8	46.0
	25 µg/mL	70.3	69.1	53.1	59.2
	12.5 µg/mL	89.1	84.2	72.2	71.3
	6.25 µg/mL	100	98.3	84.6	93.5
	3.125 µg/mL	100	100	100	100
	1.56 µg/mL	100	100	100	100
**20**	100 µg/mL	48.1	53.7	45.0	39.8
	50 µg/mL	61.3	72.5	56.2	54.6
	25 µg/mL	73.4	85.4	71.6	70.3
	12.5 µg/mL	86.7	98.9	82.1	94.1
	6.25 µg/mL	99.8	100	98.4	100
	3.125 µg/mL	100	100	100	100
	1.56 µg/mL	100	100	100	100
**21**	100 µg/mL	15.7	13.1	7.7	8.4
	50 µg/mL	22.1	19.2	16.1	17.3
	25 µg/mL	27.4	27.6	24.5	26.5
	12.5 µg/mL	42.9	38.5	38.2	37.0
	6.25 µg/mL	65.8	66.8	55.3	55.4
	3.125 µg/mL	83.2	75.7	78.1	78.8
	1.56 µg/mL	99.0	97.1	94.4	95.9

**Scheme 5:**
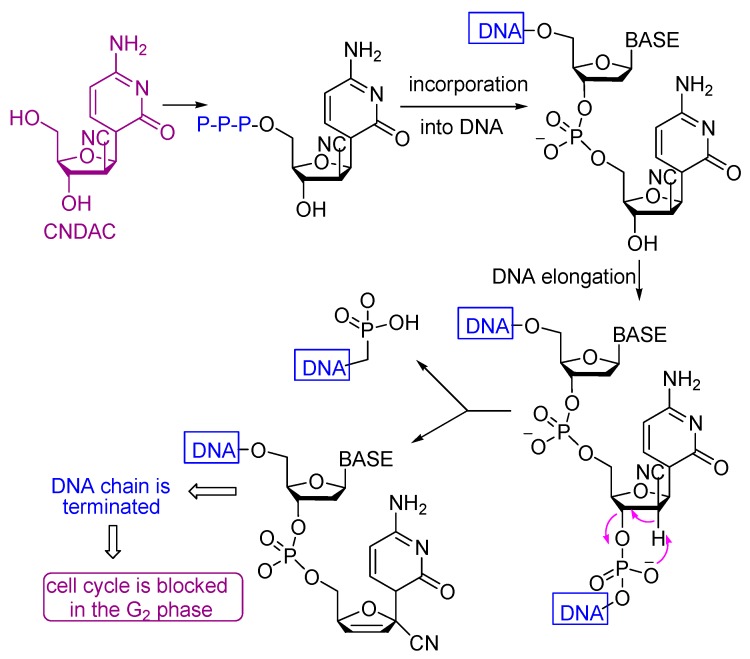
Mechanism of the antitumor action of CNDAC.

2′-C-Cyano-2′-deoxy-1-β-d-arabinopentofuranosylcytosine (CNDAC) [65] is a nucleoside analogue with a novel mechanism of action that is being evaluated in clinical trials. Incorporation of CNDAC triphosphate into DNA and extension during replication leads to single-strand breaks directly caused by β-elimination. These breaks, or the lesions that arise from further processing, cause cells to arrest in G2. The electron withdrawing effect [66] of the cyano group at the arabinose 2′-β-position increases the acidity of the 2′-α proton and facilitates a β-elimination reaction involving an oxygen of the phosphate group at the 3′-β position that leads to single strand break that affords a DNA molecule lacking a 3′-hydroxyl, which prevents its repair by ligation and leads to inhibition of the cell cycle at the G_2_ phase (Scheme 5).

**Scheme 6:**
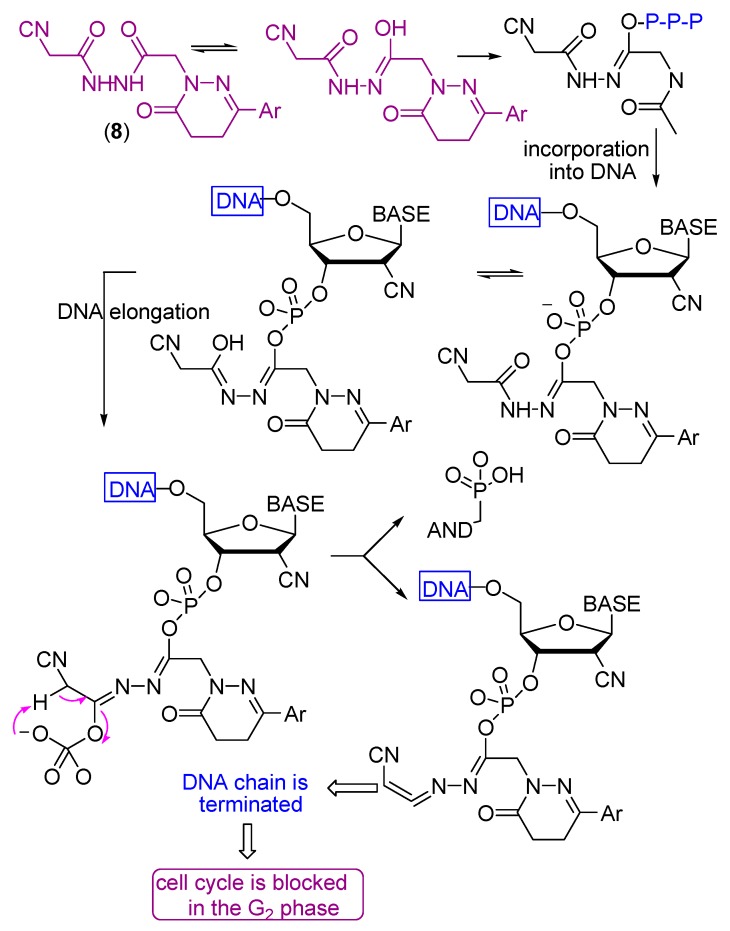
Mechanism of the antitumor action of compound **8**.

All the tested compounds showed activity, which may be due to the presence of either NH_2_, NH or OH groups which can add to any unsaturated moiety in the DNA or form a hydrogen bond with either of the nucleobases of the DNA which causes their damage to different extents. Also the presence of C≡N with α-H and β-OH enhances the cytotoxicity of compound **8** (Scheme 6). The electron withdrawing effect of the cyano group increases the acidity of the α-proton and facilitates a β-elimination reaction involving the formation of the azaallene (-C=C=N-) group. The lower activities of the other compounds compared to compound **8** is due to the absence of either α-H, β-OH or the C≡N group.

#### 2.2.2. Antioxidant Activity Using 2,2′-Azino-bis(3-ethylbenzthiazoline-6-sulfonic Acid (ABTS) Inhibition

Twelve compounds were tested for antioxidant activity as reflected in the ability to inhibit oxidation in rat brain and kidney homogenates (Table 4). Compounds **8** and **21** showed very high % inhibition, nearly equal to ascorbic acid (88.1%, 86.0% and 88.9%, respectively). Compounds **10** and **12** showed high inhibition% of 71.7 and 74.9, respectively. In addition the rest of the compounds **4**, **6**, **11**, **12**, **17**, **18**, **19** and **20** exhibited moderate to weak antioxidant activity ranging from 68.0%–47.0%.

**Table 4:** Antioxidant activity and bleomycin-dependent DNA damage caused by the tested compounds ^a^.

Comp. No.	Antioxidant Activity Absorbance	(ABTS Method) Inhibition (%)	Bleomycin Dependent DNA Damage
**4**	0.261	48.4	0.116
**6**	0.271	46.4	0.129
**8**	0.060	88.1	0.069
**9**	0.127	74.9	0.077
**10**	0.143	71.7	0.088
**11**	0.204	59.7	0.094
**12**	0.162	68.0	0.081
**17**	0.201	60.3	0.104
**18**	0.230	54.5	0.074
**19**	0.257	49.2	0.122
**20**	0.268	47.0	0.143
**21**	0.071	86.0	0.074
Control of ABTS	0.506	0	-
Ascorbic acid	0.056	88.9	0.072

^a^ All experiments were performed three times. The data are expressed as the mean-standard error of the mean (S.E.M.).

#### 2.2.3. Bleomycin-Dependent Deoxyribonucleic Acid (DNA) Damage

Bleomycin is a glycopeptide antibiotic routinely used as an antitumor agent. The bleomycin assay has been adopted for assessing the pro-oxidant effect of food antioxidants. The antitumor antibiotic bleomycin binds iron ions and DNA. The bleomycin-iron complex degrades DNA when heated with thiobarbituric acid (TBA) to yield a pink chromogenic. Upon the addition of suitable reducing agents antioxidant competes with DNA and diminishes chromogenic formation [67].

To show the mechanism of action of the tested compounds **4**, **6**, **8**, **9**, **10**, **11**, **12**, **17**, **18**, **19**, **20** and **21**, their protective activity against DNA damage induced by the bleomycin-iron complex were examined. The results (Table 4) showed that compounds **8**, **9**, **10**, **11**, **12** and **21** have the ability to protect DNA from the induced damage by bleomycin. Compound **8** showed very high protection (0.069) against DNA damage induced by the bleomycin-iron complex which is higher than ascorbic acid as a standard (0.072). Compounds **9** and **21** meanwhile showed very high protection (0.077, 0.074), respectively, against DNA damage induced by the bleomycin-iron complex which is approximately equal to ascorbic acid used as a standard (0.072). Compounds **10**, **11** and **12** showed moderate ability (0.088, 0.094 and 0.081 respectively). The rest of the compounds **4**, **6**, **17**, **18**, **19** and **20** on the other hand exhibited low activities. Thus, all the tested compounds diminish the chromogenic formation between the damage DNA and TBA.

#### 2.2.4. Structure Activity Relationship

By comparing the experimental cytotoxicity of the compounds reported in this study to their structures, the following structure activity relationships (SAR) were postulated.

- Compound **8** showed very strong activities against the four cell lines, which may be due to the presence of two NH and C≡N groups.
- Compound **9** showed very strong activities against the PC-3 and MCF-7 cell lines, and strong activity against the HePG2 and HCT-116 cell lines which may be due to the presence of NH and the oxadiazole moiety.
- Compounds **10** and **12** showed strong activity against the four cell lines, which may be due to the presence of two NH groups and the sulfur atom, which has a vacant orbital that can accept electrons.
- Compound **17** showed strong and moderate activity due to the presence of NH and OH groups.
- Compound **21** showed very strong activities against the HCT-116, PC-3 and MCF-7 cell lines, and strong activity against the HePG2 cell line which may be due to the presence of NH of the tetrazine moiety and C≡N group with α-H and β-NH.
- Compounds **4**, **6**, **11**, **18**, **19** and **20** showed either weak or moderate activities because of the absence of C≡N as in compounds **4**, **6** or the absence of α-H as in compounds **11**, **18**, **19** and **20**.

## 3. Materials and Methods

### 3.1. General Information

All melting points were measured on a Gallenkamp melting point apparatus and were uncorrected. The infrared spectra were recorded using potassium bromide disks on a Mattson FTIR spectrophotometer (Mattson, New York, NY, USA). ^1^H-NMR spectra were run at 300 MHz, on a Varian Mercury VX-300 NMR spectrometer (Bruker, Rheinstetten, Germany) using TMS as an internal standard in deuterated dimethylsulphoxide. ^13^C-NMR spectra were recorded on a Bruker spectrometer at 100 MHz. Chemical shifts δ are quoted in ppm. The mass spectra were recorded on a GCMS-QP-1000EX mass spectrometer (Shimadzu, Kyoto, Japan) at 70 e.V. All the spectral measurements were carried out at the Microanalytical Center of Cairo University, Cairo, Egypt; and the Main Defense Chemical Laboratory, Cairo, Egypt and Zagazig University, Zagazig, Egypt. The elemental analyses were carried out at the Microanalytical Center of Ain Shams University, Cairo, Egypt. The pharmaceutical activity assays were carried out at the Pharmacology Department, Faculty of Pharmacy, EL-Mansoura University, EL-Mansoura, Egypt. All reagents used in this study were commercially available. Ethyl 2-(3-(4-chloro-3-methylphenyl)-6-oxo-5,6-dihydropyridazin-1(4*H*)-yl)acetate (**1**) and 2-(3-(4-chloro-3-methylphenyl)-6-oxo-5,6-dihydropyridazin-1(4*H*)-yl)aceto-hydrazide (**3**) were prepared by previously reported procedures [25].

### 3.2. Synthesis

#### 3.2.1. *Ethyl 2-(8-(4-Chloro-3-methylphenyl)-3-thioxo-1,2,4,6,7-pentaazaspiro[4,5]deca-1,7-diene-6-yl)acetate* (**2**)

A solution of compound **1** (0.01 mol, 3.08 g) and thiosemicarbazide (0.01 mol, 0.91 g) in ethanol (30 mL) was refluxed for 6 h. The separated solid was filtered off, dried and recrystallized from ethanol as pale yellow crystals, yield 50%, mp 187–188; IR (KBr cm^−1^): 3371, 3263 (NH), 1741 (C=O); ^1^H-NMR (DMSO-*d*_6_): δ 8.58 (br.s, 1H, NH D_2_O exchangeable), 7.40–7.77 (m, 3H, Ar-H), 4.63 (s, 2H, NCH_2_), 4.14 (q, 2H, *J* = 6.9 Hz, OCH_2_), 2.98, 2.56 (2t, 4H, *J* = 8.1, 8.4 Hz, 2CH_2_), 2.37 (s, 3H, CH_3_), 1.20 (t, 3H, *J* = 6.9 Hz, CH_3_); ^13^C-NMR (DMSO-*d*_6_): 14.03, 19.60, 22.34, 26.00, 50.22, 60.76, 94.28, 128.57, 128.99, 131.32, 133.61, 135.62, 137.04, 149.60, 168.44, 181.18; MS *m*/*z* 379 (M^+^, 0.46), 308 (100), 237 (30.66), 55 (12.50). Found: C, 60.01; H, 4.80; Cl, 9.27; N, 18.65; S, 8.46%. Calcd for C_16_H_18_ClN_5_O_2_S: C, 50.59; H, 4.78; Cl, 9.33; N, 18.44; S, 8.44%.

#### 3.2.2. *2-(3-(4-Chloro-3-methylphenyl)-6-oxo-5,6-dihydropyridazin-1(4H)-yl)-N′-(2-oxoindolin-3-ylidene)-acetohydrazide* (**4**)

The acetohydrazide **3** (0.001 mol, 2.94 g) was condensed with isatin (0.001 mol, 1.47 g) in ethyl alcohol (25 mL) and a few drops of acetic acid on a water bath for 3 h. The solvent was evaporated and the reaction mixture was poured onto crushed ice. The separated solid was filtered off, dried and recrystallized from toluene as brown crystals in 2.19 g (52%); mp >168–170 °C; IR (KBr cm^−1^): 3173 (NH), 1695, 1642 (C=O), 1619 (C=N); ^1^H-NMR (DMSO-*d*_6_): δ 13.21 (br.s, 1H, NH D_2_O exchangeable), 12.57 (br.s, 1H, OH D_2_O exchangeable), 11.21 (br.s, 1H, NH D_2_O exchangeable), 6.88–7.79 (m, 7H, Ar-H), 4.67 (s, 2H, NCH_2_), 3.07, 2.62 (2t, 4H, *J* = 8.1 Hz, 2CH_2_), 2.36 (s, 3H, CH_3_); ^13^C-NMR (DMSO-*d*_6_): 19.41, 22.31, 26.08, 51.85, 122.50, 124.52, 128.14, 128.43, 128.87, 131.17, 131.61, 133.62, 134.01, 134.74, 135.55, 142.48, 149.70, 151.53, 162.41, 165.91, 169.35; MS *m*/*z* (%): 423 (M^+^, 28.14), 279 (63.03), 235 (72.03), 91 (100). Anal. Calcd for C_21_H_18_ClN_5_O_3_: C, 59.51; H, 4.28; Cl, 8.36; N, 16.52. Found: C, 59.60; H, 4.16; Cl, 8.40; N, 16.54.

#### 3.2.3. *6-(4-Chloro-3-methylphenyl)-2-(2-(3,5-dimethyl-1H-pyrazol-1-yl)-2-oxoethyl)-4,5-dihydropyridazin-3(2H)-one* (**5**)

A mixture of acetohydrazide **3** (0.01 mol, 2.94 g), acetylacetone (0.012 mol, 1.1 mL) and piperidine (few drops) was refluxed in ethanol (20 mL) for 6 h. The precipitated solid obtained was collected by filtration, dried and recrystallized from ethanol as white crystals in 2.57 g (72%), mp 129–130 °C; IR (KBr cm^−1^): 1731, 1681 (C=O); ^1^H-NMR (DMSO-*d*_6_): δ 7.40–7.78 (m, 3H, Ar-H), 6.25 (s, 1H, pyrazolo), 5.22 (s, 2H, NCH_2_), 3.05, 2.61 (2t, 4H, *J* = 8.1, 8.4 Hz, 2CH_2_), 2.49 (s, 3H, CH_3_), 2.45 (s, 3H, CH_3_), 2.21 (s, 3H, CH_3_); ^13^C-NMR (DMSO-*d*_6_): 13.45, 13.63, 19.54, 22.33, 26.03, 51.81, 111.44, 128.54, 128.90, 131.23, 133.60, 135.57, 136.97, 143.66, 149.54, 149.98, 166.07, 167.51; MS *m*/*z* 358 (M^+^, 27.99), 262 (100), 179 (13.49), 97 (39.80). Found: C, 60.10; H, 5.28; Cl, 9.79; N, 15.57%. Calcd for C_18_H_19_ClN_4_O_2_: C, 60.25; H, 5.34; Cl, 9.88; N, 15.61%.

#### 3.2.4. General Procedure for the Synthesis of Compounds **6** and **7**

A solution of the acetohydrazide **3** (0.01 mol, 2.94 g) and ethyl acetoacetate or ethyl benzoylacetate (0.01 mol) in ethanol (30 mL) was refluxed for 6 h. The separated solids were filtered off, dried and recrystallized from the proper solvent to give **6** and **7**.

*6-(4-Chloro-3-methylphenyl)-2-(2-(3-methyl-5-oxo-2H-pyrazol-1(5H)-yl)-2-oxoethyl)-4,5-dihydropyridazin-3(2H)-one* (**6**). This compound was obtained as white crystals (benzene) in 2.44 g (68%), mp 270–272 °C; IR (KBr cm^−1^): 3195 (NH), 1675, 1661, 1642 (C=O); ^1^H-NMR (DMSO-*d*_6_): δ 9.98 (s, 1H, OH D_2_O exchangeable), 7.43–7.75 (m, 4H, 3Ar-H,1H pyrazolo), 4.41 (s, 2H, NCH_2_), 3.00, 2.58 (2t, 4H, *J* = 8.1 Hz, 2CH_2_), 2.72 (s, 3H, CH_3_), 2.35 (s, 3H, CH_3_); ^13^C-NMR (DMSO-*d*_6_): 17.14, 19.54, 22.32, 26.07, 47.14, 88.57, 125.12, 128.61, 128.90, 131.28, 134.25(2 Ar-C), 135.71, 141.42, 147.14, 165.59, 197.14; MS *m*/*z* 360 (M^+^, 6.99), 308 (10.72), 262 (100), 235 (43.02), 172 (28.69), 97 (47.44). Found: C, 56.62; H, 4.79; Cl, 9.85; N, 15.49%. Calcd for C_17_H_17_ClN_4_O_3_: C, 56.59; H, 4.75; Cl, 9.83; N, 15.53%.

*6-(4-Chloro-3-methylphenyl)-2-(2-oxo-2-(5-oxo-3-phenyl-2H-pyrazol-1(5H)-yl)ethyl)-4,5-dihydropyridazin-3(2H)-one* (**7**). This compound was obtained as white crystals (ethanol) in 2.70 g (64%), mp 258–260 °C; IR (KBr cm^−1^): 3312 (NH), 1669, 1648 (C=O); ^1^H-NMR (DMSO-*d*_6_): δ 9.09 (s, 1H, OH D_2_O exchangeable), 7.40–7.75 (m, 9H, 8Ar-H,1H pyrazolo), 4.31 (s, 2H, NCH_2_), 4.24 (s, 1H, NH), 3.01, 2.55 (2t, 4H, *J* = 8.4 Hz, 2CH_2_ ring), 2.36 (s, 3H, CH_3_); ^13^C-NMR (DMSO-*d*_6_): 19.60, 22.32, 26.10, 50.18, 87.02, 124.61, 125.07, 125.86, 128.50 (2Ar-C), 128.95 (2Ar-C), 131.29, 133.75, 134.33, 134.47, 135.56, 136.86, 149.17, 149.62, 165.57, 166.83; MS *m*/*z* 422 (M^+^, 0.07), 294 (26.80), 263 (72.94), 235 (100), 55 925.48). Found: C, 62.46; H, 4.45; Cl, 8.31; N, 13.28%. Calcd for C_22_H_19_ClN_4_O_3_: C, 62.49; H, 4.53; Cl, 8.38; N, 13.25%.

#### 3.2.5. General Procedure for Synthesizing Compounds **8** and **9**

The acetohydrazide **3** (0.01 mol, 2.94 g) was fused with excess ethyl cyanoacetate at ~210 °C in an oil-bath for 40 min. Excess ethyl cyanoacetate was evaporated. The solid product was triturated with ethanol (20 mL) then filtered. The remained solid was crystallized to give **9**, and the ethanolic filtrate was poured onto crushed ice. The separated solid was filtered off, dried and recrystallized to give **8**.

*2-(3-(4-Chloro-3-methylphenyl)-6-oxo-5,6-dihydropyridazin-1(4H)-yl)-N′-(2-cyanoacet-yl)acetohydrazide* (**8**). This compound was obtained as white crystals (EtOH) in 1.59 g (46%), mp 299–301 °C; IR (KBr cm^−1^): 3182 (NH), 2212 (C≡N), 1673, 1657, 1628 (C=O); ^1^H-NMR (DMSO-*d*_6_): δ 9.89, 9.44 (2br.s, 2H, 2NH D_2_O exchangeable), 7.15–7.75 (m, 3H, Ar-H), 4.95 (s, 2H, NCH_2_CO), 4.33 (s, 2H, CH_2_CN), 2.99, 2.56 (2t, 4H, *J* = 8.4, 8.1 Hz, 2CH_2_ ring), 2.34 (s, 3H, CH_3_); ^13^C-NMR (DMSO-*d*_6_): 15.24, 22.36, 25.74, 25.74, 27.14, 114.2, 127.40, 128.96, 131.42, 132.85, 134.28 (2Ar-C), 148.45, 162.11, 170.00 (2CO); MS *m*/*z* 361 (M^+^, 2.67), 235 (100), 222 (16.17), 141 (13.85). Found: C, 53.20; H, 4.42; Cl, 9.76; N, 19.54%. Calcd for C_16_H_16_ClN_5_O_3_: C, 53.12; H, 4.46; Cl, 9.80; N, 19.36%.

*2-(5-((3-(4-Chloro-3-methylphenyl)-6-oxo-5,6-dihydropyridazin-1(4H)-yl)methylene)-4,5-dihydro-1,3,4-oxadiazol-2-yl)acetonitrile* (**9**). This compound was obtained as brown crystals (DMF) in 1.17 g (50%), mp > 300 °C; IR (KBr cm^−1^): 3207 (NH), 2206 (C≡N), 1664 (C=O); ^1^H-NMR (DMSO-*d*_6_): δ 9.99 (br.s, 1H, NH D_2_O exchangeable), 7.38–7.75 (m, 4H, 3Ar-H, 1HC=), 4.41 (s, 2H, CH_2_CN), 3.01, 2.58 (2t, 4H, *J* = 8.4 Hz, 2CH_2_ ring), 2.35 (s, 3H, CH_3_); ^13^C-NMR (DMSO-*d*_6_): 14.03, 19.60, 23.92, 32.51, 60.75, 120.14, 128.60, 128.93, 129.65, 131.27, 134.25, 135.57, 149.76, 158.84, 172.18, 175.26; MS *m*/*z* 343 (M^+^, 12.07), 123 (20.53), 55 (100). Found: C, 55.67; H, 4.12; Cl, 10.26; N, 20.54%. Calcd for C_16_H_14_ClN_5_O_2_: C, 55.90; H, 4.10; Cl, 10.31; N, 20.37%.

#### 3.2.6. Another Method for Synthesizing Compound **8**

A mixture of acetohydrazide **3** (0.01 mol, 2.94 g), ethyl cyanoacetate (0.01 mol, 1.13 mL) and drops of piperidine was refluxed in ethanol (20 mL) for 6 h. The separated solid after cooling was filtered off, dried and recrystallized from ethanol to give compound **8**.

#### 3.2.7. Another Method for Synthesizing Compound (**9**)

Compound **8** was refluxed in ethanol for 3 h. The reaction mixture was evaporated and the separated solid was filtered off, dried and recrystallized to give **9**.

#### 3.2.8. General Procedure for Synthesizing Compounds **10**–**13**

To a stirred suspension of finely powdered potassium hydroxide (0.02 mol, 1.12 g) in dry DMF (10 mL) compound **8** or **9** (0.01 mol) was added. The resulted mixture was cooled at 10 °C in an ice bath and then carbon disulfide (0.50 mL, 0.01 mol) was added slowly over the course of 10 min. After complete addition, stirring of the reaction mixture was continued for additional 2 h. Then dimethylsulfate or dibromoethane (0.01 mol) was added to the mixture while cooling (~15 °C) and stirring for 1 h. then poured onto crushed ice, the resulting precipitate was filtrated off, dried and crystallized from the proper solvent to give compounds **10**–**13** respectively.

*N′-(2-(3-(4-Chloro-3-methylphenyl)-6-oxo-5,6-dihydropyridazin-1(4H)-yl)acetyl)-2-cyano-3,3-bis(methylthio)acrylohydrazide* (**10**). This compound was obtained as pale brown crystals (methanol) in 3.25 g (70%), mp > 300 °C; IR (KBr cm^−1^): 3220 (NH), 2204 (C≡N), 1675 (C=O); ^1^H-NMR (DMSO-*d*_6_): δ 9.97 (br.s, 2H, 2NH D_2_O exchangeable), 7.43–7.75 (m, 3H, Ar-H), 4.40 (s, 2H, CH_2_N), 3.04, 2.55 (2t, 4H, *J* = 8.1, 8.4 Hz, 2CH_2_ ring), 2.89 (s, 6H, 2SCH_3_), 2.35 (s, 3H, CH_3_); ^13^C-NMR (DMSO-*d*_6_): 14.24, 19.60 (2CH_3_), 22.38, 26.05, 57.14, 78.57, 117.14, 128.93, 129.09, 131.42, 132.85, 134.79, 135.69, 146.60, 162.85, 165.89, 170.00, 175.71; MS *m*/*z* 465 (M^+^, 0.00), 450 (0.20), 418 (0.37), 71 (80.44), 57 (100). Found: C, 48.67; H, 4.39; Cl, 7.78; N, 15.13; S, 13.58%. Calcd for C_19_H_20_ClN_5_O_3_S_2_: C, 48.97; H, 4.33; Cl, 7.61; N, 15.03; S, 13.76%.

*2-(5-((3-(4-Chloro-3-methylphenyl)-6-oxo-5,6-dihydropyridazin-1(4H)-yl)methylene)-4,5-dihydro-1,3,4-oxadiazol-2-yl)-3,3-bis(methylthio)acrylonitrile* (**11**). This compound was obtained as brown crystals (acetic acid) in 3.57 g (80%), mp > 300 °C; IR (KBr cm^−1^): 3327 (NH), 2207 (C≡N), 1642 (C=O), 1627 (C=N); ^1^H-NMR (DMSO-*d*_6_): δ 9.99 (br.s, 1H, NH D_2_O exchangeable), 6.93–8.12 (m, 4H, 3Ar-H, 1HC = oxadiazole), 3.00, 2.54 (2t, 4H, *J* = 8.4 Hz, 2CH_2_ ring), 2.88 (s, 6H, 2SCH_3_), 2.34 (s, 3H, CH_3_); ^13^C-NMR (DMSO-*d*_6_): 14.03, 17.32 (2CH_3_), 23.83, 31.42, 67.97, 76.32, 116.21, 127.40, 128.85, 129.63, 131.29, 134.54, 135.64, 146.51, 158.21, 171.64, 171.90, 173.91; MS *m*/*z* 447 (M^+^, 0.18), 432 (0.32), 353 (0.25), 71 (81.61), 57 (100). Found: C, 50.83; H, 4.96; Cl, 7.98; N, 15.13; S, 14.22%. Calcd for C_19_H_18_ClN_5_O_2_S_2_: C, 50.94; H, 4.05; Cl, 7.91; N, 15.63; S, 14.32%.

*N'-(2-(3-(4-Chloro-3-methylphenyl)-6-oxo-5,6-dihydropyridazin-1(4H)-yl)acetyl)-2-cyano(1,3-dithiolan-2-ylidene)acetohydrazide* (**12**). This compound was obtained as pale brown crystals (dioxane) in 3.00 g (65%), mp ˃ 300 °C; IR (KBr cm^−1^): 3436 (NH), 2206 (C≡N), 1656, 1646 (C=O), 1625 (C=N); ^1^H-NMR (DMSO-*d*_6_): δ 10.20 (br.s, 1H, NH D_2_O exchangeable), 9.98 (s, 1H, NH D_2_O exchangeable), 7.38–7.75 (m, 3H, 3Ar-H), 4.41 (s, 2H, CH_2_CO), 3.15 (s, 4H, SCH_2_-CH_2_S), 3.00, 2.55 (2t, 4H, *J* = 8.4 Hz, 2CH_2_ ring), 2.35 (s, 3H, CH_3_); ^13^C-NMR (DMSO-*d*_6_): 14.67, 25.97, 26.10, 34.28(2CH_2_S), 52.80, 81.40, 118.13, 127.83, 128.93, 130.32, 132.68, 135.49, 135.56, 149.55, 164.73, 165.86, 169.87, 179.57; MS *m*/*z* 463 (M^+^, 6.10), 368 (31.25), 297 (21.59), 270 (100), 185 (63.94) 55 (53.34). Found: C, 49.26; H, 3.89; Cl, 7.63; N, 15.16; S, 13.85%. Calcd for C_19_H_18_ClN_5_O_3_S_2_: C, 49.19; H, 3.91; Cl, 7.64; N, 15.09; S, 13.82%.

*2-(5-((3-(4-Chloro-3-methylphenyl)-6-oxo-5,6-dihydropyridazin-1(4H)-yl)methylene)-4,5-dihydro-1,3,4-oxadiazol-2-yl)-2-(1,3-dithiolan-2-ylidene)acetonitrile* (**13**). This compound was obtained as brown crystals (dioxane) in 3.00 g (68%), mp 119–120 °C; IR (KBr cm^−1^): 3200 (NH), 2214 (C≡N), 1677 (C=O); ^1^H-NMR (DMSO-*d*_6_): δ 10.00 (br.s, 1H, NH D_2_O exchangeable), 7.43–7.95 (m, 4H, 3Ar-H, 1HC= oxadiazole), 4.41 (s, 4H, 2SCH_2_), 2.89, 2.60 (2t, 4H, *J* = 8.1 Hz, 2CH_2_ ring), 2.34 (s, 3H, CH_3_); ); ^13^C-NMR (DMSO-*d*_6_): 19.57, 26.10, 30.79, 35.80 (2CH2S), 68.57, 72.85, 112.85, 128.62, 128.93, 130.00, 131.42, 134.54, 135.63, 144.28, 162.34, 166.51, 174.28, 184.28; MS *m*/*z* 445 (M^+^, 0.57), 235 (76.08), 77 (100). Found: C, 51.15; H, 3.60; Cl, 7.92; N, 15.69; S, 14.37%. Calcd for C_19_H_16_ClN_5_O_2_S_2_: C, 51.17; H, 3.62; Cl, 7.95; N, 15.70; S, 14.38%.

#### 3.2.9. General Procedure for Synthesizing Compounds **14** and **15**

To suspension of potassium hydroxide (0.01 mol, 0.56 g) in dry DMF (10 mL) compounds **8** or **9** (0.01 mol) was added during stirring, phenyl isothiocyanate (0.01 mol, 1.20 mL) was dropped slowly to the reaction mixture. After complete of addition, stirring of the reaction mixture was continued for 5 h. and dimethyl sulfate (0.01 mol, 0.94 mL) was added. The reaction mixture was stirred for 2 h. then, poured onto crushed ice. The resulting precipitate was filtered off, dried and recrystallized from the proper solvent to give **14** or **15**.

*N′-(2-(3-(4-chloro-3-methylphenyl)-6-oxo-5,6-dihydropyridazin-1(4H)-yl)acetyl)-2-cyano-3-(methylthio)-3-(phenylamino)acrylohydrazide* (**14**). This compound was obtained as brown crystals (ethanol) in 6.3 g (72%), mp > 300 °C; IR (KBr cm^−1^): 3182 (NH), 2203 (C≡N), 1673 br (C=O broad); ^1^H-NMR (DMSO-*d*_6_): δ 10.59, 10.27, 10.07 (three br.s, 3H, 3NH D_2_O exchangeable), 6.92–8.71 (m, 8H, Ar-H), 4.41 (s, 2H, CH_2_CO), 3.00, 2.75 (2t, 4H, *J* = 8.4 Hz, 2CH_2_ ring), 2.58 (s, 3H, SCH_3_), 2.36 (s, 3H, CH_3_); ^13^C-NMR (DMSO-*d*_6_): 14.24, 15.30, 23.60, 26.10, 53.18, 70.63, 115.21, 116.50 (2Ar-C), 118.35, 127.41, 128.85, 129.72 (2Ar-C), 137.03, 135.71, 131.41, 132.63, 144.56, 146.44, 162.54, 165.62, 170.31, 177.14; MS *m*/*z* 511 (M^+^, 0.00), 496 (0.72), 439 (15.10), 425 (100), 263 (10.30), 235 (29.40), 135 (44.93), 63 (46.51). Found: C, 56.57; H, 4.58; Cl, 6.77; N, 16.63; S, 6.35%. Calcd for C_24_H_23_ClN_6_O_3_S: C, 56.41; H, 4.54; Cl, 6.94; N, 16.45; S, 6.28%.

*2–5-((3-(4-Chloro-3-methylphenyl)-6-oxo-5,6-dihydropyridazin-1(4H)-yl)methylene)-4,5-dihydro-1,3,4-oxadiazol-2-yl)-3-(methylthio)-3-(phenylamino)acrylonitrile* (**15**). This compound was obtained as brown crystals (acetic acid) in 3.88 g (79%), mp > 300 °C; IR (KBr cm^−1^): 3333, 3211 (NH), 2202 (C≡N), 1714 (C=O); ^1^H-NMR (DMSO-*d*_6_): δ 8.62, 7.57 (2br.s, 2H, 2NH D_2_O exchangeable), 7.27–7.73 (m, 8H, Ar-H), 6.96 (s,1H, =CH), 3.00, 2.67 (2t, 4H, *J* = 8.1 Hz, 2CH_2_ ring), 2.41 (s, 3H, SCH_3_), 2.34 (s, 3H, CH_3_); ^13^C-NMR (DMSO-*d*_6_): 14.03, 15.90, 22.82, 32.51, 65.28, 68.56, 115.87, 116.01 (2Ar-C), 118.27, 127.37, 128.64, 129.52 (2Ar-C), 130.59, 132.71, 136.25, 135.57, 144.41, 146.20, 156.28, 170.98, 172.19, 173.64; Found: C, 58.30; H, 4.28; Cl, 7.22; N, 16.97; S, 6.47%. Calcd for C_24_H_21_ClN_6_O_2_S: C, 58.47; H, 4.29; Cl, 7.19; N, 17.05; S, 6.50%.

#### 3.2.10. *2-(3-(4-Chloro-3-methylphenyl)-6-oxo-5,6-dihydropyridazin-1(4H)-yl)-N-(3-cyano-4,6-dimethyl-2-oxopyridin-1(2H)-yl)acetamide* (**16**)

A mixture of compound **8** (0.01 mol, 3.61 g), acetylacetone (0.012 mol, 1.1 mL) and piperidine (few drops) in ethanol (20 mL) was refluxed for 6 h. The obtained solid was collected by filtration, dried and recrystallized from ethanol as brown crystals in 2.6 g (63%), mp > 300 °C; IR (KBr cm^−1^): 3180 (NH), 2214 (C≡N), 1677, 1657, 1642 (C=O); ^1^H-NMR (DMSO-*d*_6_): δ 9.99 (br.s, 1H, NH D_2_O exchangeable), 7.38–7.75 (m, 4H, 3Ar-H, 1HC=), 4.41 (s, 2H, CH_2_N), 3.01, 2.58 (2t, 4H, *J* = 8.4, 8.1 Hz, 2CH_2_ ring), 2.35 (s, 6H, 2CH_3_), 2.28 (s, 3H, CH_3_)); ^13^C-NMR (DMSO-*d*_6_): 15.7 (2 CH_3_), 19.45, 22.32, 26.08, 54.28, 108.57, 114.28, 115.71, 125.11, 128.57, 131.42, 132.85, 134.24, 135.71 (2Ar-C), 146.45, 152.85, 158.57, 165.61, 166.47; MS *m*/*z* 425 (M^+^, 0.00), 427 (M + 2, 1.31), 322 (3.80), 149 (15.82), 111 (25.78), 97 (40.66), 57 (100). Found: C, 59.12; H, 4.89; Cl, 8.40; N, 16.59%. Calcd for C_21_H_20_ClN_5_O_3_: C, 59.23; H, 4.73; Cl, 8.32; N, 16.44%.

#### 3.2.11. General Procedure for Synthesizing Compounds **17** and **18**

A mixture of compounds **8** and/or **9** (0.01 mol) and salicylaldehyde, (1.07 g, 0.01 mol) in ethanol (30 mL) containing ammonium acetate (0.3 g) was heated under reflux for 0.5 h. The solvent was evaporated and the obtained solid product was filtered off and recrystallized from the proper solvent.

*N′-(2-(3-(4-Chloro-3-methylphenyl)-6-oxo-5,6-dihydropyridazin-1(4H)-yl)acetyl)-2-cyano-3-(2-hydroxyphenyl)acrylohydrazide* (**17**). This compound was obtained as brown crystals (acetic acid) in 3.16 g (68%), mp > 300 °C; IR (KBr cm^−1^): 3414 (OH), 3188 (NH), 2206 (C≡N), 1671, 1653, 1628 (C=O broad); ^1^H-NMR (DMSO-*d*_6_): δ 9.99 (br.s, 2H, 2NH D_2_O exchangeable), 8.41 (s, 1H, 1HC=C), 7.38–7.75 (m, 7H, 3Ar-H), 7.18 (br.s, 1H, OH D_2_O exchangeable), 4.41 (s, 2H, CH_2_N), 3.01, 2.58 (2t, 4H, *J* = 8.4, 8.1 Hz, 2CH_2_ ring), 2.34 (s, 3H, CH_3_); ^13^C-NMR (DMSO-*d*_6_): 19.55, 22.32, 26.06, 54.28, 111.12, 115.90, 116.10, 118.80, 119.50, 127.14 (2Ar-C), 128.90, 129.28, 131.42, 132.85, 135.57, 138.12, 145.71, 154.28, 158.57, 161.42, 165.62, 171.42; MS *m*/*z* 465 (M^+^, 0.22), 238 (90.24), 164 (100). Found: C, 59.22; H, 4.25; Cl, 7.77; N, 15.12%. Calcd for C_23_H_20_ClN_5_O_4_: C, 59.29; H, 4.33; Cl, 7.61; N, 15.03%.

*2-(5-((3-(4-chloro-3-methylphenyl)-6-oxo-5,6-dihydropyridazin-1(4H)-yl)methylene)-4,5-dihydro-1,3,4-oxadiazol-2-yl)-3-(2-hydroxyphenyl)acrylonitrile* (**18**). This compound was obtained as brown crystals (ethanol) in 3.39 g (76%), mp > 300 °C; IR (KBr cm^−1^): 3419 (OH), 3200 (NH), 2207 (C≡N), 1671 (C=O); ^1^H-NMR (DMSO-*d*_6_): δ 9.97 (br.s, 1H, NH D_2_O exchangeable), 7.45–7.75 (m, 9H, 7Ar-H, 1HC=C, 1OH), 2.99, 2.72 (2t, 4H, *J* = 8.4, 8.1 Hz, 2CH_2_ ring), 2.35 (s, 3H, CH_3_); ^13^C-NMR (DMSO-*d*_6_): 19.55, 26.08, 28.99, 67.14, 101.42, 115.71 (2Ar-C), 116.01, 121.42, 128.57, 128.59 (2Ar-C), 128.89, 134.23, 134.52, 135.57, 137.04, 140.80, 149.74, 155.87, 157.14, 165.61, 166.48; MS *m*/*z* 447 (M^+^, 0.19), 337 (0.78), 111 (34.25), 85 (56.42), 71 (69.22), 57 (100). Found: C, 61.57; H, 3.99; Cl, 7.89; N, 15.68%. Calcd for C_23_H_18_ClN_5_O_3_: C, 61.68; H, 4.05; Cl, 7.92; N, 15.64%.

#### 3.2.12. General Procedure for Synthesizing Compounds **19** and **20**

To a mixture of compounds **8** or **9** (0.01 mol), *p*-anisaldehyde (0.01 mol, 1.22 g) and malononitrile (0.01 mol, 0.66 g) in ethanol (30 mL), a few drops of piperidine were added. The reaction mixture was heated under reflux for 3 h. The solid product which formed was collected by filtration while hot and recrystallized from the appropriate solvent to give **19** and **20**.

*N-(6-Amino-3,5-dicyano-4-(4-methoxyphenyl)-2-oxopyridin-1(2H)-yl)-2-(3-(4-chloro-3-methylphenyl)-6-oxo-5,6-dihydropyridazin-1(4H)-yl)acetamide* (**19**). This compound was obtained as brown crystals (acetic acid) in 3.47 g (64%), mp > 300 °C; IR (KBr cm^−1^): 3457, 3271, 3178 (NH_2_, NH), 2206 (C≡N), 1676, 1642 (C=O), 1623 (C=N); ^1^H-NMR (DMSO-*d*_6_): δ 10.00 (br.s, 1H, NH D_2_O exchangeable), 9.87 (br.s, 2H, NH_2_ D_2_O exchangeable), 7.11–7.97 (m, 7H, Ar-H), 4.47 (s, 2H, CH_2_N), 3.87 (s, 3H, OCH_3_), 2.98, 2.55 (2t, 4H, *J* = 8.4 Hz, 2CH_2_ ring), 2.35 (s, 3H, CH_3_); ^13^C-NMR (DMSO-*d*_6_): 19.54, 22.20, 26.08, 54.28, 55.68, 75.71, 113.78 (2Ar-C), 114.50 (2C≡N), 114.60, 122.85, 125.11 (2Ar-C), 128.59, 128.90, 131.79, 132.85, 134.28, 135.71, 145.71, 152.85, 161.42, 164.28, 165.71, 170.00, 191.30; MS *m*/*z* 543 (M^+^, 0.16), 235 (100), 221 (29.80), 57 (38.20). Found: C, 59.59; H, 4.09; Cl, 6.48; N, 18.10%. Calcd for C_27_H_22_ClN_7_O_4_: C, 59.62; H, 4.08; Cl, 6.52; N, 18.02%.

*5-Amino-2-((3-(4-chloro-3-methylphenyl)-6-oxo-5,6-dihydropyridazin-1(4H)-yl)methylene)-7-(4-methoxyphenyl)-3,7-dihydro-2H-[1,3,4]oxadiazolo[3,2-a]pyridine-6,8-dicarbonitrile* (**20**). This compound was obtained as brown crystals (EtOH) in 3.05 g (58%), mp 198–200 °C; IR (KBr cm^−1^): 3448, 3178 (NH_2_, NH), 2206 (C≡N), 1674 (C=O); ^1^H-NMR (DMSO-*d*_6_): δ 8.40 (br.s, 1H, NH D_2_O exchangeable), 6.95–7.71 (m, 7H, Ar-H), 5.07 (s, 1H, =CH), 4.41 (s, 1H, CH), 3.74 (s, 3H, OCH_3_), 3.02, 2.60 (2t, 4H, *J* = 8.4, 8.1 Hz, 2CH_2_ ring), 2.72 (br.s, 2H, NH_2_ D_2_O exchangeable), 2.72 (s, 3H, CH_3_); ^13^C-NMR (DMSO-*d*_6_): 15.49, 22.90, 32.48, 37.51, 55.92, 56.80, 57.79, 72.94, 114.62, (2Ar-C), 117.25 (2C≡N), 127.54, 128.82, 130.21 (2Ar-C), 131.24, 132.30, 134.72, 136.37, 136.92, 144.05, 146.48, 154.28, 160.85, 166.47, 170.03; MS *m*/*z* 527 (M^+^, 2.58), 480 (37.37), 368 (25.21), 278 (41.76), 263 (52.49), 235 (100), 121 (43.93), 55 (60.27). Found: C, 61.46; H, 3.99; Cl, 6.80; N, 18.59%. Calcd for C_27_H_22_ClN_7_O_3_: C, 61.42; H, 4.20; Cl, 6.72; N, 18.57%.

#### 3.2.13. *2-(6-((3-(4-Chloro-3-methylphenyl)-6-oxo-5,6-dihydropyridazin-1(4H)-yl) methyl)-1,2-dihydro-1,2,4,5-tetrazin-3-yl)acetonitrile* (**21**)

A mixture of compound **8** (0.01 mol, 3.61 g) and hydrazine hydrate (0.01 mol, 0.50 mL) in ethanol (20 mL) was refluxed for 3 h. The separated solid was filtered off, dried and recrystallized from benzene as brown crystals in 2.82 g (79%), mp 168–170 °C; IR (KBr cm^−1^): 3312 (NH), 2199 (C≡N), 1667 (C=O); ^1^H-NMR (DMSO-*d*_6_): δ 10.47, 9.07 (two br.s, 2H, 2NH D_2_O exchangeable), 7.35–7.71 (m, 3H, Ar-H), 4.31 (s, 2H, CH_2_N), 4.26 (s, 2H, CH_2_CN), 3.01, 2.55 (2t, 4H, *J* = 8.1, 8.4 Hz, 2CH_2_ ring), 2.36 (s, 3H, CH_3_); ^13^C-NMR (DMSO-*d*_6_): 15.63, 18.20, 22.60, 29.31, 49.38, 115.97, 127.75, 128.82, 131.20, 132.11, 136.50, 136.72, 146.72, 162.37, 163.40 (2C=N); MS *m*/*z* 357 (M^+^, 27.99), 262 (100), 172 (21.93), 97 (39.80). Found: C, 53.99; H, 4.12; Cl, 9.98; N, 27.37%. Calcd for C_16_H_16_ClN_7_O: C, 53.71; H, 4.51; Cl, 9.91; N, 27.40%.

#### 3.2.14. *2-(5-((3-(4-Chloro-3-methylphenyl)-6-oxo-5,6-dihydropyridazin-1(4H)-yl)methyl)-4H-benzo[g]- [1,3,4,6]-thiatriazocin-2-yl)acetonitrile* (**22**)

A mixture of compound **8** (3.61 g, 0.01 mol) and 2-amino thiophenol (0.01 mol, 1.25 mL) in DMF (20 mL) was refluxed for 3 h. The reaction mixture was poured on water. The separated solid was filtered off, dried and recrystallized from ethanol as brown crystals in 1.89 g (42%), mp > 300 °C; IR (KBr cm^−1^): 3328, 3182 (NH), 2205 (C≡N), 1660 (C=O), 1612 (C=N); ^1^H-NMR (DMSO-*d*_6_): δ 6.40–7.93 (m, 7H, Ar-H), 5.44 (br.s, 1H, NH D_2_O exchangeable), 4.39 (s, 2H, CH_2_N), 2.95 (s, 2H, CH_2_CN), 2.86, 2.71 (2t, 4H, *J* = 8.4, 8.1 Hz, 2CH_2_ ring), 2.37 (s, 3H, CH_3_); ^13^C-NMR (DMSO-*d*_6_): 15.24, 19.99, 23.40, 26.89, 50.61, 115.89, 122.85, 124.30, 127.00, 127.54 (2Ar-C), 128.86, 130.53 (2Ar-C), 131.75, 136.48, 136.90, 146.52, 153.97, 154.01, 162.53, 163.76; MS *m*/*z* 450 (M^+^, 0.06), 358 (27.99), 262 (100) 172 (21.93), 97 (39.80). Found: C, 58.77; H, 4.28; Cl, 7.67; N, 18.73; S, 7.205. Calcd for C_22_H_19_ClN_6_OS: C, 58.60; H, 4.25; Cl, 7.86; N, 18.64; S, 7.11%.

### 3.3. Pharmacological Activity

#### 3.3.1. Cytotoxicity Assay

The cytotoxic activity of twelve compounds was tested against four human tumor cell lines namely: hepatocellular carcinoma (liver) HePG-2, colon cancer HCT-116, human (prostate) cancer cell line PC3, and mammary gland (breast) MCF-7. The cell lines were obtained from the ATCC via the Holding Company for Biological Products and Vaccines (VACSERA, Cairo, Egypt). 5-Fluorouracil was used as a standard anticancer drug for comparison. The reagents used were RPMI-1640 medium, MTT, DMSO and 5-fluorouracil (Sigma Co., St. Louis, MO, USA), and Fetal Bovine Serum (GIBCO, Paisley, UK).

##### MTT Assay

The different cell lines [68,69] mentioned above were used to determine the inhibitory effects of compounds on cell growth using the MTT assay. This colorimetric assay is based on the conversion of the yellow tetrazolium bromide (MTT) to a purple formazan derivative by mitochondrial succinate dehydrogenase in viable cells. The cells were cultured in RPMI-1640 medium with 10% fetal bovine serum. Antibiotics added were 100 units/mL penicillin and 100 µg/mL streptomycin at 37 °C in a 5% CO_2_ incubator. The cell lines were seeded [70] in a 96-well plate at a density of 1.0 × 10^4^ cells/well at 37 °C for 48 h under 5% CO_2_ incubator. After incubation the cells were treated with different concentration of compounds and incubated for 24 h. After 24 h of drug treatment, 20 µL of MTT solution at 5 mg/mL was added and incubated for 4 h. Dimethyl sulfoxide (DMSO) in volume of 100 µL is added into each well to dissolve the purple formazan formed. The colorimetric assay is measured and recorded at absorbance of 570 nm using a plate reader (EXL 800, BioTech, Winoosky, VT, USA). The relative cell viability in percentage was calculated as (A_570_ of treated samples/A_570_ of untreated sample) × 100.

#### 3.3.2. Antioxidant Assay

##### ABTS Method

For each of the investigated compounds [71,72,73] ABTS solution (60 µM, 2 mL) was added to MnO_2_ suspension (25 mg/mL, 3 mL), all prepared in aqueous phosphate buffer solution (pH 7, 0.1 M, 5 mL). The mixture was shaken, centrifuged, filtered and the absorbance of the resulting green blue solution (ABTS radical solution) at 734 nm was adjusted to approx. *ca.* 0.5. Then, a solution (50 µL, 2 mM) of the tested compound in spectroscopic grade MeOH/phosphate buffer (1:1) was added. The absorbance was measured and the reduction in color intensity was expressed as inhibition percentage. l-ascorbic acid was used as standard antioxidant (positive control). Blank sample was run without ABTS and using MeOH/phosphate buffer (1:1) instead of the tested compounds. Negative control was run with ABTS and MeOH/phosphate buffer (1:1) only.

##### Bleomycin—Dependent DNA Damage Assay

To the reaction mixtures [74,75] in a final volume of 1.0 mL, the following reagents were added: DNA (0.2 mg/mL), bleomycin sulfate (0.05 mg/mL), FeCl_3_ (0.025 mM), magnesium chloride (5 mM), KH_2_PO_4_–KOH buffer pH 7.0 (30 mM), and ascorbic acid (0.24 mM) or the test fractions diluted in MeOH to give a concentration of (0.1 mg/mL). The reaction mixtures were incubated in a water bath at 37 °C for 1 h. At the end of the incubation period, 0.1 mL of ethylenediaminetetraacetic acid (EDTA) (0.1 M) was added to stop the reaction (the iron-EDTA complex is unreactive in the bleomycin assay). DNA damage was assessed by adding 1 mL 1% (*w*/*v*) thiobarbituric acid (TBA) and 1 mL of 25% (*v*/*v*) hydrochloric acid followed by heating in a water-bath maintained at 80 °C for 15 min. The chromogen formed was extracted into 1-butanol, and the absorbance was measured at 532 nm.

## 4. Conclusions

In this work, the acetohydrazide reacted with excess ethyl cyanoacetate to afford cyanoacetylacetohydrazide and oxadiazolylacetonitrile derivatives. The latter compounds reacted with different nitrogen and carbon nucleophiles to give bis(methylthio), dithiolan-2-ylidene, methylthio-3-phenylamino, oxopyridine, hydroxyphenyl and tetrazine derivatives. Some of the newly prepared compounds were tested *in vitro* against a panel of four human tumor cell lines and also as antioxidants. Almost all of the tested compounds showed satisfactory activity. Compounds **8** and **21** showed activity towards MCF-7 and PC-3 cell line nearly equal to the 5-flurouracil, respectively. Also they showed very high % inhibition nearly equal to the ascorbic acid. Hence, they could be potential drugs candidate for cancer treatment.

## Supplementary Materials

Supplementary materials can be accessed at: http://www.mdpi.com/1420-3049/21/2/155/s1.
