# Supplementary material for: Utilization of Cyanoacetohydrazide and Oxadiazolyl Acetonitrile in the Synthesis of Some New Cytotoxic Heterocyclic Compounds
Source: Molecules. 2016 Jan 29;21(2):155. doi: 10.3390/molecules21020155 (PMC6273806; doi:10.3390/molecules21020155)
Supplement: Supplementary file 1 [file molecules-21-00155-s001.pdf]

Supplementary Materials: Utilization of Cyanoacetohydrazide and Oxadiazolyl Acetonitrile in Synthesis of Some New Cytotoxic Heterocyclic Compounds

Soheir A. Shaker and Magda I. Marzouk

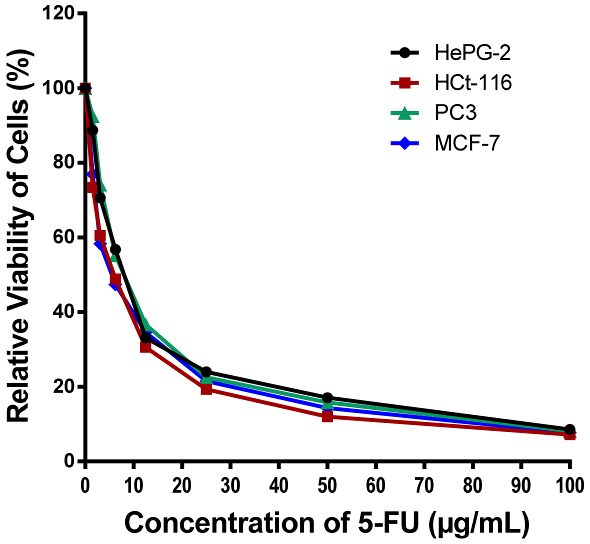

Figure S1. Relative viability of cells (%) against concentration of 5-FU.

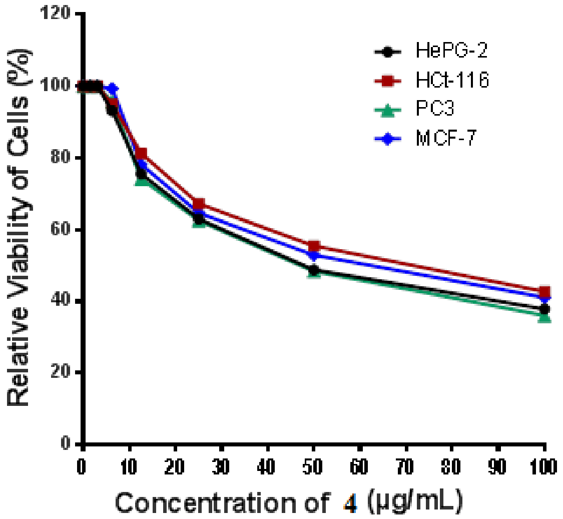

Figure S2. Relative viability of cells (%) against concentration of compound **4**.

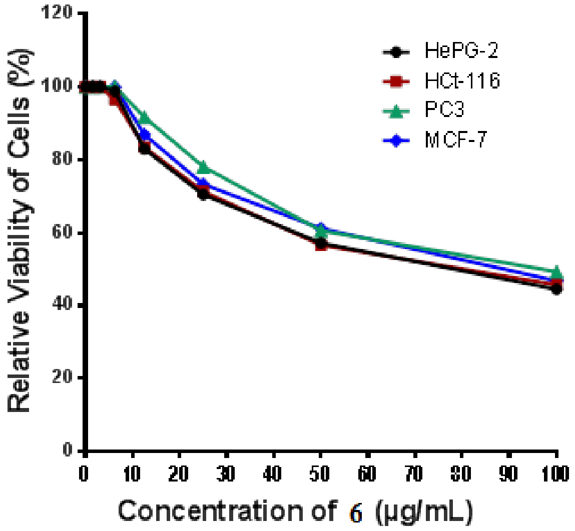

Figure S3. Relative viability of cells (%) against concentration of compound **6**.

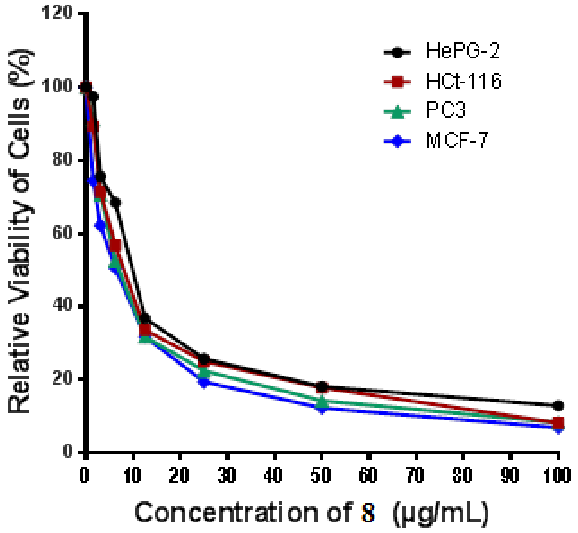

Figure S4. Relative viability of cells (%) against concentration of compound **8**.

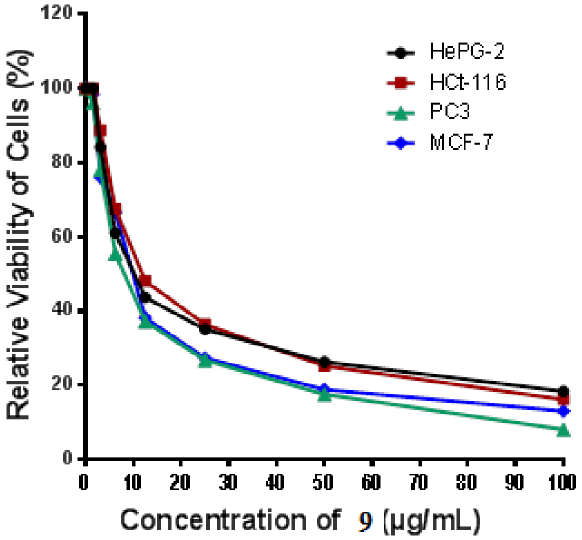

Figure S5. Relative viability of cells (%) against concentration of compound **9**.

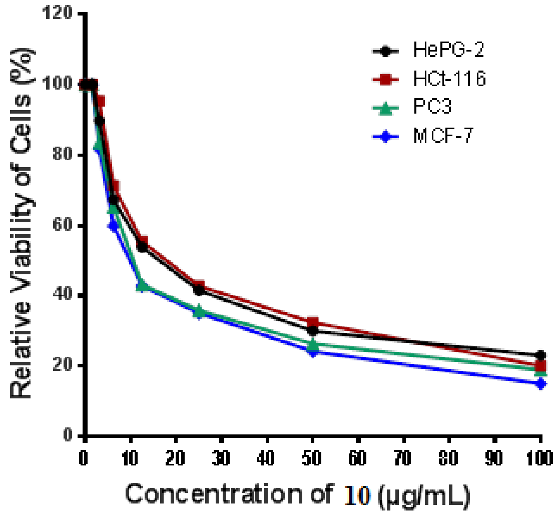

Figure S6. Relative viability of cells (%) against concentration of compound **10**.

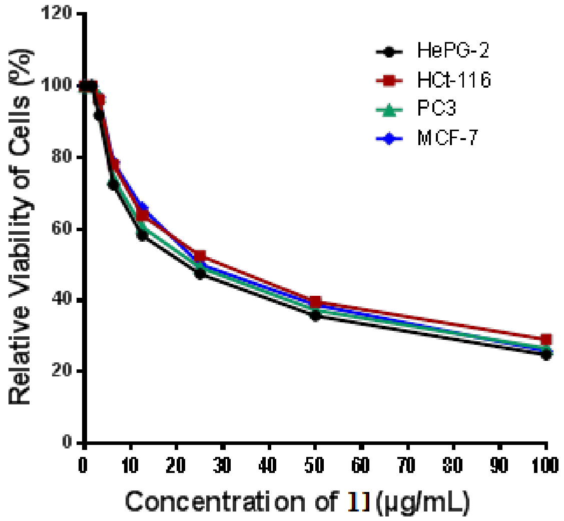

Figure S7. Relative viability of cells (%) against concentration of compound **11**.

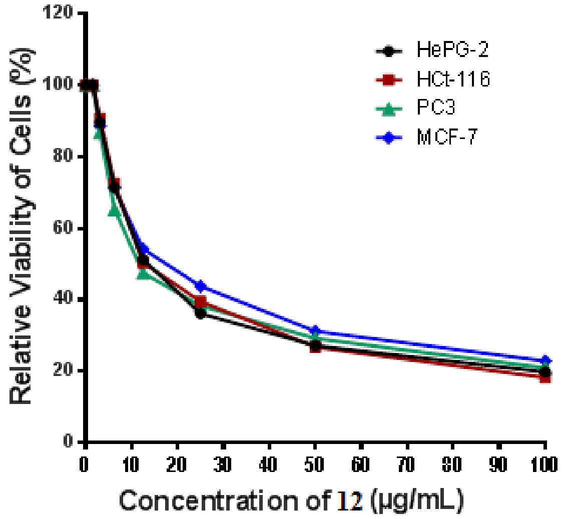

Figure S8. Relative viability of cells (%) against concentration of compound **12**.

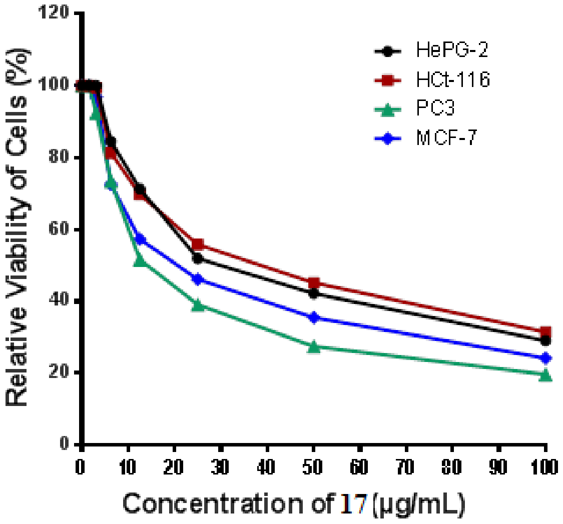

Figure S9. Relative viability of cells (%) against concentration of compound **17**.

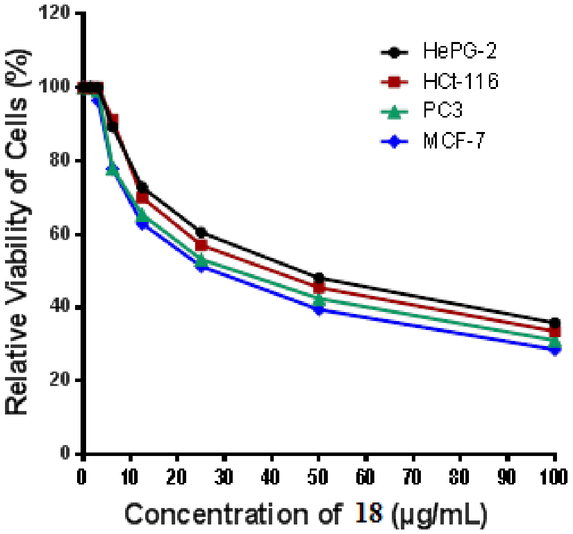

Figure S10. Relative viability of cells (%) against concentration of compound **18**.

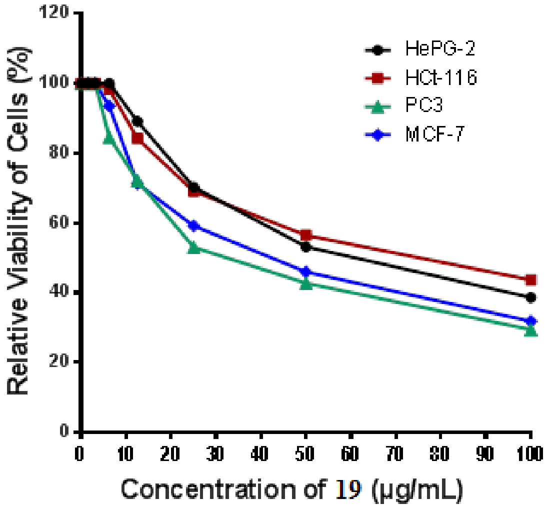

Figure S11. Relative viability of cells (%) against concentration of compound **19**.

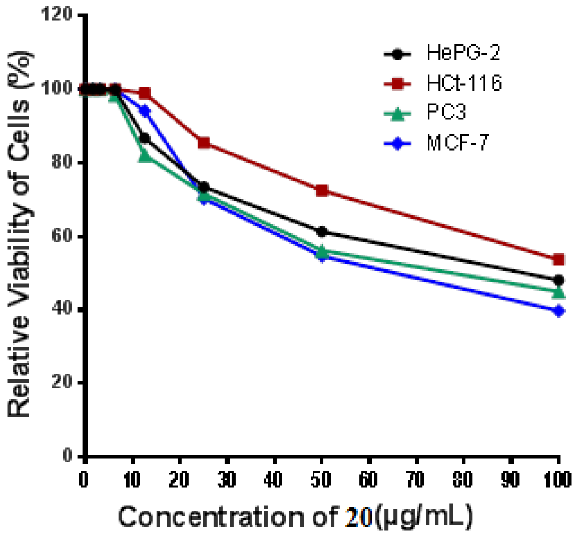

Figure S12. Relative viability of cells (%) against concentration of compound **20**.

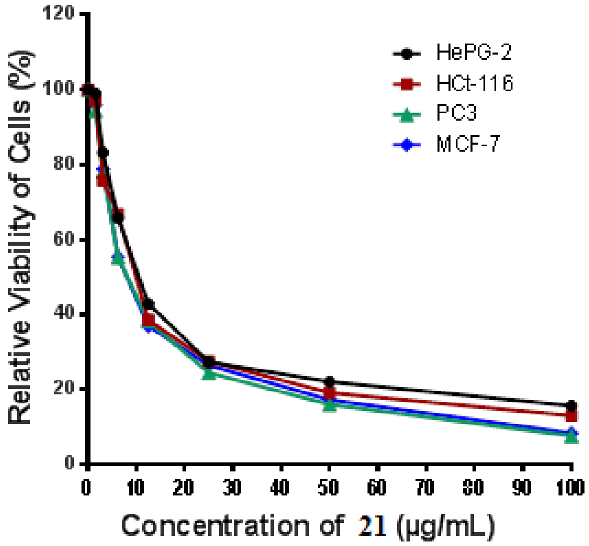

Figure S13. Relative viability of cells (%) against concentration of compound **21**.
